# Mental health symptoms and associated factors for general population at the stable, recurrence, and end-of-emergency stages of the COVID-19 pandemic: a repeated national cross-sectional study

**DOI:** 10.1017/S2045796025100243

**Published:** 2025-10-14

**Authors:** Shu Wang, Yuan Zhang, Wei Ding, Yao Meng, Huiting Hu, Yuguang Guan, Xianwei Zeng, Zhenhua Liu, Fangang Meng, Minzhong Wang, Jianguo Zhang

**Affiliations:** 1 Department of Neurosurgery, Beijing Tiantan Hospital, Capital Medical University, Beijing, China; 2 Neonatal Center, Beijing Children’s Hospital, Capital Medical University, National Center for Children’s Health, Beijing, China; 3 Department of Public Health, Liaocheng People’s Hospital, Liaocheng, China; 4 Department of Neurology, The First Affiliated Hospital of Shandong First Medical University, Jinan, China; 5 Department of Neurology, Heze Mudan People’s Hospital, Heze, China; 6 Department of Neurosurgery, SanBo Brain Hospital, Capital Medical University, Beijing, China; 7 Department of Neurosurgery, Qilu Hospital of Shandong University, Jinan, China; 8 Sleep Medicine Center, Shandong Provincial Hospital Affiliated to Shandong First Medical University, Jinan, China; 9 Beijing Neurosurgical Institute, Beijing Tiantan Hospital, Capital Medical University, Beijing, China; 10 Beijing Key Laboratory of Neurostimulation, Beijing, China; 11 Department of Neurology, Shandong Provincial Hospital Affiliated to Shandong First Medical University, Jinan, China

**Keywords:** anxiety, COVID-19, depression, insomnia, mental health, pandemic, post-traumatic stress disorder, risk factor

## Abstract

**Aims:**

The COVID-19 pandemic exacerbated psychological distress, but limited information is available on the shifts in mental health symptoms and their associated factors across different stages. This study was conducted to more reliably estimate shifts in mental health impacts and to identify factors associated with symptoms at different pandemic stages.

**Methods:**

We performed a national repeated cross-sectional study at stable (2021), recurrence (2022), and end-of-emergency (2023) stages based on representative general national population with extensive geographic coverage. Anxiety, depression, post-traumatic stress disorder (PTSD) and insomnia symptoms were evaluated by GAD-7, PHQ-9, IES-R and ISI scales, respectively, and their associated factors were identified via multivariable linear regression.

**Results:**

Generally, 42,000 individuals were recruited, and 36,218, 36,097 and 36,306 eligible participants were included at each stage. The prevalence of anxiety, depression and insomnia symptoms increased from 13.7–16.4% at stable to 17.3–22.2% at recurrence and decreased to 14.5–18.6% at end of emergency, while PTSD symptom continuously increased from 5.1% to 7.6% and 9.2%, respectively (all significant, *P* < 0.001). Common factors associated with mental health symptoms across all stages included centralized quarantine, frontline work and residence in initially widely infected areas. Centralized quarantine was linked to anxiety, depression, PTSD and insomnia during the stable, recurrence and end-of-emergency stages. Frontline workers exhibited higher risks of anxiety, depression and insomnia throughout these stages. Individuals in initially widely infected areas were more likely to experience depression and PTSD, particularly during the stable and recurrence stages. Stage-specific risk factors were also identified. Lack of outdoor activity was associated with anxiety, depression and insomnia during the stable and recurrence stages. Residents in high-risk areas during the recurrence stage correlated with increased anxiety and insomnia. Suspected infection was tied to anxiety and insomnia in the recurrence and end-of-emergency stages, while the death of family or friends was linked to PTSD during recurrence and to depression, PTSD and insomnia at the end-of-emergency stage.

**Conclusions:**

Mental health symptoms increased when pandemic recurred, and could remain after end-of-emergency, requiring prolonged interventions. Several key factors associated with mental symptoms and their variations were identified at different pandemic stages, suggesting different at-risk populations.

## Introduction

Coronavirus disease 2019 (COVID-19) quickly emerged in late 2019 and was quickly designated a global pandemic (Chen *et al.*, 2020; Stein *et al.*, 2022). As an acute global emergency, COVID-19 is very contagious (Tan *et al.*, 2023) and leads to multisystem symptoms, which can even be life threatening for some patients (Parotto *et al.*, 2023; Wiersinga *et al.*, 2020), with more than 770 million cases globally over the past 3 years (Marks and Gulick, 2023; World Health Organization, 2023). The latest Global Burden of Disease Study 2021 estimates that COVID-19 is the leading cause of disability-adjusted life-years globally (GBD 2021 Diseases and Injuries Collaborators, 2024), with 15.9 million excess deaths from 2020 to 2021(GBD 2021 Demographics Collaborators, 2024). In May 2023, the World Health Organization (WHO) announced that COVID-19 ‘no longer constitutes a public health emergency of international concern’, considering that it has not been an unusual or unexpected event (Harris, 2023). However, COVID-19 has been and will continue evolving to influence global health through long symptoms, new mutations, and frequent recurrence, which necessitates the attention of global health (El-Shabasy *et al.*, 2022; Yisimayi *et al.*, 2024).

During the COVID-19 pandemic, multiple stress factors, such as worries about exposure and infection, social isolation and physical inactivity, and unavailable psychosocial support, could exacerbate psychological distress (Shi *et al.*, 2020; Wang *et al.*, 2020, 2021) and impact mental health, such as anxiety, depression, sleep problems, post-traumatic stress disorder (PTSD) and other symptoms (Alimoradi *et al.*, 2021; Cénat *et al.*, 2021; Salanti *et al.*, 2022). This implication has been of global concern, as growing evidence has revealed an increased mental health burden (COVID-19 Mental Disorders Collaborators, 2021; Kola *et al.*, 2021; Prime *et al.*, 2020). However, major gaps and concerns remain regarding the shifts in mental health impacts and associated factors during different pandemic periods, and limited information is available on post-acute mental health symptoms (Penninx *et al.*, 2022; Pirkis *et al.*, 2021; Raina *et al.*, 2021). Additionally, the results from many studies have shown substantial heterogeneity among their study populations and times (Hossain *et al.*, 2020; Penninx *et al.*, 2022) and have limited methodological quality due to small sample sizes and convenience sampling, as well as unclear representativeness and generalizability(Cénat *et al.*, 2021; Salanti *et al.*, 2022). To more reliably estimate shifts in mental health impacts and to identify factors associated with symptoms at different pandemic stages, we conducted this large-sample multicentre study with a repeated cross-sectional design at 3 representative stages (stable, recurrence, and end-of-emergency) in the general Chinese population with our previous experience during the initial COVID-19 wave (Wang *et al.*, 2020) and return-to-work period (Wang *et al.*, 2021; Zhang *et al.*, 2020). This study can also provide timely references for the post-COVID-19 era and future potential pandemics and recurrence, helping in more appropriately mediating health policies and identifying and protecting individuals at risk and promoting long-term resilience; thus, providing important information for policy makers, practicing clinicians, researchers and other stakeholders (Penninx *et al.*, 2022).

## Materials and methods

### Study design and sampling process

This repeated cross-sectional study was conducted through 3 surveys from 2021 to 2023. The ethics committee of Shandong Provincial Hospital Affiliated to Shandong First Medical University approved this study. All participants provided online or oral informed consent. This study followed the 1964 Helsinki Declaration and its later amendments and reports according to the Statement of Reporting of Observational studies in Epidemiology (STROBE; von Elm *et al.*, 2014). The surveys were anonymous, and the confidentiality of the data was ensured.

**Fig. 1.**
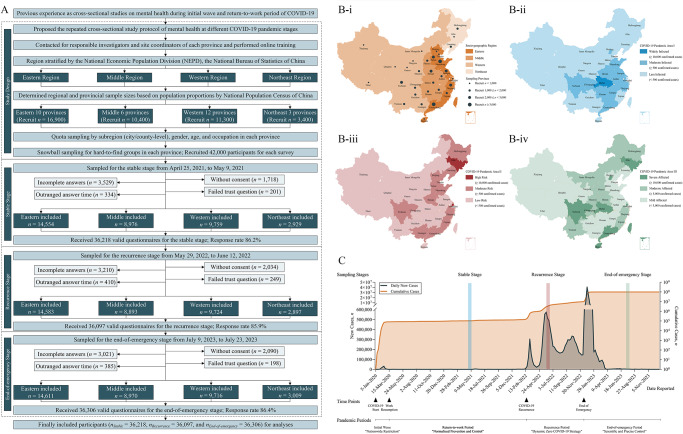
Flow diagram showing the study procedure (A), sketch map showing the region divisions (B), and timeline showing the COVID-19 stages (**C**). COVID-19, coronavirus disease 2019; NEPD, National Economic Population Division. Region division was classified based on socio-geographical characteristics or the influence of COVID-19 on regional features and infection risk at different pandemic stages, refers to **Supplementary Table 1** for detailed regional division for provinces; socio-geographic region was stratified based on NEPD, the National Bureau of Statistics for the normal period; COVID-19 pandemic area I was stratified according to cumulative confirmed cases between January 2020 and March 2020 (initial wave, 2020) and data from the National Health Commission, China; COVID-19 pandemic area II was stratified according to cumulative confirmed cases between March 2022 and May 2022 (recurrence, 2022) and data from the National Health Commission, China; and COVID-19 pandemic area III was stratified according to cumulative confirmed cases between January 2020 and December 2022 (end-of-emergency, 2023).

The study procedure is shown in Fig. 1. Based on our previous experience during the COVID-19 epidemic (Wang *et al.*, 2020, 2021; Zhang *et al.*, 2020), we proposed the current study protocol and applied a region-stratified population-based quota combined with a snowball sampling strategy to recruit a representative national sample, which was previously established when random sampling was inappropriate during the epidemic (Wang *et al.*, 2020, 2021; Zhang *et al.*, 2020). First, we contacted the investigators and site coordinators of all 31 provinces of mainland China and performed unified online training. According to the National Economic Population Division (NEPD) of the National Bureau of Statistics (NBS; National Bureau of Statistics of People’s Republic of China, 2011), these provinces were stratified into four socio-geographic regions: eastern, middle, western and northeast. Second, the required sample sizes of each region and affiliated provinces were determined based on the population proportions of the National Population Census (NPC) 2020 (Office of the Leading Group of the State Council for the Seventh National Population Census, 2020). Third, we calculated the required quotas to recruit individuals with representative characteristics by further stratifying the proportions of subregions (at the city/county level), genders, ages and occupations for each sampling province. After that, investigators were required to invite individuals with these specific characteristics. The invitations were sent online, by telephone, via posters or person to person. When an individual agreed for participation and was verified to have required characteristics, he/she was marked as ‘recruited’ and sent with surveys. Snowball sampling was used as a supplement for some hard-to-find groups (e.g., elderly individuals) when necessary, which encouraged participants to introduce others with the required characteristics to the investigators. A nominal payment (lucky draw) was set as a recruitment incentive for all participants. Finally, the same procedure was repeated triply at the stable (25 April 2021 to 9 May 2021), recurrence (29 May 2022 to 12 June 2022) and end-of-emergency (9 July 2023 to 23 July 2023) COVID-19 pandemic stages. Participants could participate in one or more different surveys but were allowed to answer only once for the same survey.

Pandemic periods classification, key time points and health policies were collected from epidemic reports by the National Health Commission of China (NHC; www.nhc.gov.cn). Specifically, from late 2019 to March 2020, China experienced initial epidemic wave and applied ‘nationwide restriction’ policy. This initial wave was basically controlled from February 2020 and the ‘normalized prevention and control’ during this stable stage. However, the epidemic recurrent from March 2022, during which the ‘dynamic zero’ policy were applied. In December 2022, the Chinese government declared the end of the emergency of the COVID-19 epidemic and announced the ‘scientific and precise control’ (Fig. 1).

Additionally, the NHC database and the WHO dashboard (World Health Organization, 2023) were queried to stratify the infection risks of these provinces during different pandemic periods in exploring its potential influences on mental health at different pandemic stages. Pandemic area I (initial wave) was stratified according to cumulative confirmed cases between January 2020 and March 2020 in capturing regional characteristics of the initial waves; pandemic area II (recurrence) was stratified according to cumulative confirmed cases between March 2022 and May 2022 to classified risks of the first COVID-19 recurrence waves; and pandemic area III (end-of-emergency, summary of the pandemic) was stratified according to cumulative confirmed cases between January 2020 and December 2022 in summarizing the entire pandemic situation until the end of emergency. The detailed regional divisions are shown in **Supplementary Table 1**.

### Study population

This study assessed the mental health of the general population. Considering ethical issues and measurement scales, only adults (≥18 years) were included. The target sample size for recruitment was calculated in PASS software version 2021 (NCSS LLC) for multiple (3) comparisons of proportions (Chow *et al.*, 2008), in which the statistical power was set to 90.0% and the overall α = 0.05 (Bonferroni adjusted α = 0.0167). Based on previous studies (Wang *et al.*, 2020, 2021; Xiong *et al.*, 2020; Zhang *et al.*, 2020), the proportion of mental health symptoms was estimated to be 15%, and the acceptable margin of differences was set to 1%. When the dropout rate was set to 20%, the dropout-inflated enrolment sample size was 41,864 for each stage. Considering the convenience of calculating the required quotas, we increased the sample size to 42,000 for each survey stage.

For these participants, 5,782 (1,718 without consent, 3,529 with incomplete answers, 201 with failed trust questions and 334 with outrange answer times), 5,903 (2,034 without consent, 3,210 with incomplete answers, 249 with failed trust questions and 410 with outrange answer times) and 5,694 (2,090 without consent, 3,021 with incomplete answers, 198 with failed trust questions and 385 with outrange answer times) invalid questionnaires were excluded for the stable, recurrence, and end-of-emergency stages, respectively. The events per variable (EPV) for the final eligible participants also met the requirements for logistic regression (all EPVs ≥ 10) (Chow *et al.*, 2008) in identifying factors associated with symptoms (Table 1).

**Table 1.** Socio-demographic characteristics, activity and work/study status, relevant experiences, and psychological interventions of all included participants at different COVID-19 pandemic stages (*n*_Stable_ = 36,218, *n*_Recurrence_ = 36,097 and *n*_End-of-emergency_ = 36,306)

	No. (%)			
Subject	Stable stage (sampled 2021)	Recurrence stage (sampled 2022)	End-of-emergency stage (sampled 2023)	*P* value
Overall	36,218 (100.0)	36,097 (100.0)	36,306 (100.0)	
(Response rate), recruited participants *n* = 42,000	(86.2)	(85.9)	(86.4)	0.11
Region division^a^				
Socio-geographic region (NEPD, normal period)				0.89
Eastern region	14,554 (40.2)	14,583 (40.4)	14,611 (40.2)	
Middle region	8,976 (24.8)	8,893 (24.6)	8,970 (24.7)	
Western region	9,759 (26.9)	9,724 (26.9)	9,716 (26.8)	
Northeast region	2,929 (8.1)	2,897 (8.1)	3,009 (8.3)	
COVID-19 pandemic area I (initial wave, 2020)				
Widely-infected area (≥10,000 confirmed cases)	1,463 (4.0)	1,465 (4.1)	1,471 (4.1)	>0.999
Moderate-infected area (≥500 confirmed cases)	20,098 (55.5)	20,005 (55.4)	20,130 (55.4)	
Less-infected area (<500 confirmed cases)	14,657 (40.5)	14,627 (40.5)	14,705 (40.5)	
COVID-19 pandemic area II (recurrence, 2022)				
High-risk area (≥10,000 confirmed cases)	NA	1,395 (3.9)	1,419 (3.9)	0.93
Moderate-risk area (≥500 confirmed cases)	NA	19,434 (53.8)	19,567 (53.9)	
Low-risk area (<500 confirmed cases)	NA	15,268 (42.3)	15,320 (42.2)	
COVID-19 pandemic area III (end-of-emergency, 2023)				
Severe affected area (≥10,000 confirmed cases)	NA	NA	9,273 (25.5)	NA
Moderate affected area (≥5,000 confirmed cases)	NA	NA	10,814 (29.8)	
Mild affected area (<5,000 confirmed cases)	NA	NA	16,219 (44.7)	
Socio-demographic characteristic				
Gender				0.65
Male	18,556 (51.2)	18,392 (51.0)	18,611 (51.3)	
Female	17,662 (48.8)	17,705 (49.0)	17,695 (48.7)	
Age, years				0.47
18−34	9,940 (27.4)	9,881 (27.4)	10,027 (27.6)	
35−49	10,424 (28.8)	10,327 (28.6)	10,338 (28.5)	
50−64	9,668 (26.7)	9,549 (26.5)	9,751 (26.9)	
≥65	6,186 (17.1)	6,340 (17.5)	6,190 (17.0)	
Place of residence				0.74
Urban	19,656 (54.3)	19,682 (54.5)	19,707 (54.3)	
Rural	16,562 (45.7)	16,415 (45.5)	16,599 (45.7)	
Education level				
Less than college	28,332 (78.2)	28,274 (78.3)	28,622 (78.8)	0.10
College degree or higher	7,886 (21.8)	7,823 (21.7)	7,684 (21.2)	
Marriage status				0.61
Unmarried	6,960 (19.2)	6,929 (19.2)	7,023 (19.3)	
Married	26,403 (72.9)	26,397 (73.1)	26,393 (72.7)	
Divorced/Widowed	2,855 (7.9)	2,771 (7.7)	2,890 (8.0)	
History of chronic diseases				0.72
Yes	3,255 (9.0)	3,258 (9.0)	3,299 (9.1)	
No	31,835 (87.9)	31,777 (88.0)	31,916 (87.9)	
Unknown	1128 (3.1)	1062 (3.0)	1091 (3.0)	
History of psychiatric disorders				0.20
Yes	413 (1.1)	428 (1.2)	429 (1.2)	
No	34,708 (95.9)	34,610 (95.9)	34,704 (95.6)	
Unknown	1097 (3.0)	1059 (2.9)	1173 (3.2)	
Occupation				0.57
Students, full-time	1,723 (4.8)	1,681 (4.7)	1,691 (4.7)	
Technicians and associate professionals	3,600 (9.9)	3,679 (10.2)	3,613 (10.0)	
Government and clerical support workers	3,229 (8.9)	3,226 (8.9)	3,249 (8.9)	
Social and life service workers	9,516 (26.3)	9,534 (26.4)	9,684 (26.7)	
Agricultural, forestry and fishery workers	7,006 (19.3)	7,071 (19.6)	7,097 (19.5)	
Production and manufacture workers	8,989 (24.8)	8,884 (24.6)	8,793 (24.2)	
Other unclassified occupations	105 (0.3)	97 (0.3)	103 (0.3)	
Freelance or inoccupation	2,050 (5.7)	1,925 (5.3)	2,076 (5.7)	
Yearly family income, CNY^b^				0.86
<40,000	7,497 (20.7)	7,537 (20.9)	7,557 (20.8)	
40,000−99,999	22,725 (62.7)	22,690 (62.9)	22,803 (62.8)	
≥100,000	5,996 (16.6)	5,870 (16.2)	5,946 (16.4)	
Activity and work/study status				
Outside activity/once				<0.001*
1−7 days	19,230 (53.1)	7,837 (21.7)^c^	22,947 (63.2)^c,d^	
8−14 days	10,982 (30.3)	11,904 (33.0)^c^	10,061 (27.7)^c,d^	
15−29 days	3,908 (10.8)	8,920 (24.7)^c^	2,532 (7.0)^c,d^	
≥30 days	2,098 (5.8)	7,436 (20.6)^c^	766 (2.1)^c,d^	
Work/study status				<0.001*
On-site work/study	22,391 (61.8)	10,001 (27.7)^c^	28,847 (79.5)^c,d^	
Off-site work/study	7,301 (20.2)	16,581 (45.9)^c^	4,810 (13.2)^c,d^	
Not back to work/study	6,526 (18.0)	9,515 (26.4)^c^	2,649 (7.3)^c,d^	
Experience related to COVID-19				
Current COVID-19 identity				<0.001*
Current infected	402 (1.1)	4,322 (12.0)^c^	2,257 (6.2)^c,d^	
Previous infected	3,349 (9.2)	6,291 (17.4)^c^	24,821 (68.4)^c,d^	
Suspect infected	557 (1.5)	5,299 (14.7)^c^	2,783 (7.7)^c,d^	
Not infected	31,910 (88.2)	20,185 (55.9)^c^	6,445 (17.7)^c,d^	
Frontline workers during COVID-19^a,b^				<0.001*
Yes	6,038 (16.7)	6,698 (18.6)^c^	7,976 (22.0)^c,d^	
No	30,180 (83.3)	29,399 (81.4)^c^	28,330 (78.0)^c,d^	
Experience of hospitalization for COVID-19				<0.001*
Yes	2,710 (7.5)	4,515 (12.5)^c^	8,176 (22.5)^c,d^	
No	33,508 (92.5)	31,582 (87.5)^c^	28,130 (77.5)^c,d^	
Experience of quarantine during COVID-19				
Centralized	3,788 (10.5)	6,989 (19.4)^c^	10,125 (27.9)^c,d^	<0.001*
At home	6,219 (17.2)	10,294 (28.5)^c^	16,997 (46.8)^c,d^	
None	26,211 (72.3)	18,814 (52.1)^c^	9,184 (25.3)^c,d^	
Families/friends hospitalization related to COVID-19				<0.001*
Yes	5,487 (15.1)	9,111 (25.2)^c^	16,435 (45.3)^c,d^	
No	30,731 (84.9)	26,986 (74.8)^c^	19,871 (54.7)^c,d^	
Families/friends death related to COVID-19				<0.001*
Yes	837 (2.3)	3,731 (10.3)^c^	5,350 (14.7)^c,d^	
No	35,381 (97.7)	32,366 (89.7)^c^	30,956 (85.3)^c,d^	
Psychological intervention during COVID-19				
Psychological intervention during COVID-19				<0.001*
Yes	9,266 (25.6)	10,678 (29.6)^c^	12,594 (34.7)^c,d^	
Public psychological education only	7,997 (86.3)	9,196 (86.1)	10,803 (85.8)	0.52
With individual counselling	1269 (13.7)	1482 (13.9)	1,791 (14.2)	
No	26,952 (74.4)	25,419 (70.4)^c^	23,712 (65.3)^c,d^	

COVID-19, coronavirus disease 2019; NEPD, National Economic Population Division, by the National Bureau of Statistics, China; NA, not applicable.

a Region division was classified based on different socio-geographical characteristics or COVID-19 influences in reflecting regional features and infection risks at different pandemic stages, refers to **eTable 1** for detailed region division for provinces; Socio-geographic region was stratified based on NEPD, the National Bureau of Statistics, China, for normal period; COVID-19 pandemic area I was stratified according to cumulative confirmed cases between January 2020 to March 2020 (initial wave, 2020), data from the National Health Commission, China; COVID-19 pandemic area II was stratified according to cumulative confirmed cases between March 2022 to May 2022 (recurrence, 2022), data from the National Health Commission, China; COVID-19 pandemic area III was stratified according to cumulative confirmed cases between January 2020 to December 2022 (end-of-emergency, 2023). ^b^As of September 2nd, 2024, 1 CNY = 0.139 USD, the stratification could be regarded as ‘low-income family’, ‘moderate-income family’ and ‘high-income family’ based on standard of the National Bureau of Statistics, China. ^a,b,^Frontline workers indicate individuals who directly participated in the control of COVID-19, serving with the potential for direct or indirect exposure to COVID-19 patients or infectious materials. *P < 0.01 (Pearson’s *χ^2^* test). ^c^(Bonferroni) adjusted *P* < 0.05 comparing with the Stable stage (post hoc *z*-test for pairwise comparisons, adjusted by Bonferroni correction). ^d^(Bonferroni) adjusted *P* < 0.05 comparing with the Recurrence stage (post hoc *z*-test for pairwise comparisons, adjusted by Bonferroni correction).

### Measurements and covariates

The same questionnaire design was applied for the 3 surveys. Participants preferred to answer online via WJX links (Ranxing LLC) with an IP address restriction to prevent repeated answers from the same person in the survey. For those who could not finish the online survey, telephone interviews read by investigators were provided with the same content. Additionally, participants were asked to answer only one type of survey to avoid duplication. A guiding webpage or oral introduction of the informed consent was provided prior to the survey. The participants were informed about their free decision to participate or not participate, provided informed consent or not, and could terminate the survey at any time.

The self-designed survey consisted of 4 sections and required approximately 15 minutes to complete. The first section collected socio-demographic information, including sex, age (division according to previous epidemiological studies [Huang *et al.*, 2019; Wang *et al.*, 2020, 2021; Zhang *et al.*, 2020] for comparisons), place of residence (urban vs rural), education level, marital status, history of chronic disease and psychiatric disorders, occupation (classified by the NPC 2020), and yearly family income. The second section asked pandemic-related questions about current activity (outside activity frequency) and work/study status (on-site, off-site or not), experience related to COVID-19 (current identity, frontline workers or not, experience of hospitalization and quarantine, and hospitalization and death of family/friends), and psychological interventions during COVID-19 (with or without; and public psychological education only, i.e., provided by governments, societies, communities and others, which was delivered once or scattered times without specialized guiding, or with individual counselling, i.e., provided by professional psychologists with systematic terms and individualized treatments).

The third section included 4 standardized screening scales, including the Generalized Anxiety Disorder-7 scale (GAD-7, scores from 0-21, Cronbach’s α coefficient = 0.93)(Zhang *et al.*, 2021), Patient Health Questionnaire-9 (PHQ-9, scores from 0-27, Cronbach’s α coefficient = 0.90)(Wang *et al.*, 2014; Zhang *et al.*, 2013), Impact of Events Scale-Revised (IES-R, scores from 0-88, Cronbach’s α coefficient = 0.95)(Creamer *et al.*, 2003; Wu and Chan, 2003), and Insomnia Severity Index (ISI, scores from 0-28, Cronbach’s α coefficient = 0.91)(Chung *et al.*, 2011; Thorndike *et al.*, 2011), which measured anxiety, depression, PTSD, and insomnia symptoms, respectively. These scales are all validated Chinese versions and have been widely used in previous epidemiological investigations (Chen *et al.*, 2023; Lai *et al.*, 2020; Shi *et al.*, 2020; Xiong *et al.*, 2020). Higher scores on these scales indicate more severe symptoms. In the present study, the cut-off scores for detecting symptoms were ≥10, ≥10, ≥33 and ≥15, and scores ≥15, ≥15, ≥37 and ≥22 indicated severe symptoms for the GAD-7, PHQ-9, IES-R and ISI, respectively. These cut-off values were determined according to Chinese norms and previous studies of the Chinese population (Chen *et al.*, 2023; Wang *et al.*, 2020, 2021; Xiong *et al.*, 2020; Zhang *et al.*, 2020), which has been widely recognized and further reviewed by a consensus of neuropsychologists.

For the fourth section, two trust test questions were designed: ‘I answered truthfully (yes or no)’ and ‘What is ten plus ten?’ Surveys without consent, with incomplete answers, with a failure of any trust question, or with an outrange of time (i.e., <1 min or >2 hours) were regarded as invalid questionnaires.

### Statistical analysis

Categorical variables are reported as numbers and percentages. The prevalence of symptoms was calculated, and 95% confidence intervals (CIs) were determined by exact binomial methods. Pearson’s *x*^2^ test was used to compare categorical variables. For pairwise comparisons of multiple groups, post hoc *z-*tests were applied after adjusting by Bonferroni correction. Considering the incomplete answers were missing randomly and variables in regression analysis, all analyses were based on complete data (Graham, 2009).

Logistic regression was used to explore potential factors (such as region divisions, socio-demographic characteristics, activity and work/study status, experience related to COVID-19, and psychological interventions) associated with symptoms. All factors with significance in the univariable unadjusted logistic analyses, which might convey important information, were then entered into multivariable logistic regression (backward) to adjust for confounding effects of other variables in the model. The contrast was used as an indicator of the subgroup with the lowest prevalence to explore potential risk factors. The adjusted odds ratios (aORs), 95% CIs and *P* values of the risk factors are provided. Additionally, multicollinearity diagnostics tested by variance inflation factors were verified with < 10 in the final model, suggesting the independence of these factors.

In this study, all the statistical tests were two-sided, and the significance level was set at α = 0.05. All analyses were performed in SPSS software version 27 (IBM Crop) and R software version 4 (R Foundation), and the figures were drawn using GraphPad Prism version 10 (GraphPad Software LLC).

## Results

### Socio-demographic characteristics

Data from a total of 36,218, 36,097, and 36,306 participants were included in the final analysis at the stable, recurrence and end-of-emergency stages, respectively, with a response rate comparable to 85.9–86.4%. Generally, 93.9–95.0% and 5.0–6.1% of the participants were recruited from quota and snowball sampling; and 87.6–89.0% and 11.0–12.1% of them were surveyed online or through telephone, respectively. Participants recruited from quota or snowball sampling, as well as surveyed online or through telephone were compared with nonsignificant difference in characteristics or outcomes, confirming their equivalence (all *P* > 0.05). Baseline characteristics of the included participants (subregions, genders, ages and occupations) were compared with the designed quotas and the NPC 2020, and no significant difference was revealed (all *P* > 0.05), suggesting that the included participants had sufficient representativeness of the general population and inconsequential influences of the missing data.

No significant difference was found in the distribution of regional populations among these 3 surveys, and their socio-demographic characteristics were comparable (Table 1), indicating good comparability. Among these participants at each survey stage, 18,392–18,611 (51.0–51.3%) participants were reported as male, aged 18–87 years (IQR 32–58, the same for 3 surveys), and 19,656–19,707 (54.3–54.5%) participants were urban residents. Most participants had an educational level less than college (28,274–28,622 [78.2–78.8%]) and were married (26,393–26,403 [72.7–73.1%]). A significant difference was found in pandemic-related variables. Increased participants have previously infected (3,349 [9.2%] vs 6,291 [17.4%] vs 24,821 [68.4%]), served as frontline workers (6,038 [16.7%] vs 6,698 [18.6%] vs 7,976 [22.0%]), experienced hospitalization (2,710 [7.5%] vs 4,515 [12.5%] vs 8,176 [22.5%]), quarantine (centralized, 3,788 [10.5%] vs 6,989 [19.4%] vs 10,125 [27.9%]), and at home, 6,219 [17.2%] vs 10,294 [28.5%] vs 16,997 [46.8%]), and had family/friends’ hospitalization (5,487 [15.1%] vs 9,111 [25.2%] vs 16,435 [45.3%]) and death (837 [2.3%] vs 3,731 [10.3%] vs 5,350 [14.7%]) related to COVID-19 from the stable stage to recurrence and end-of-emergency stages.

**Fig. 2.**
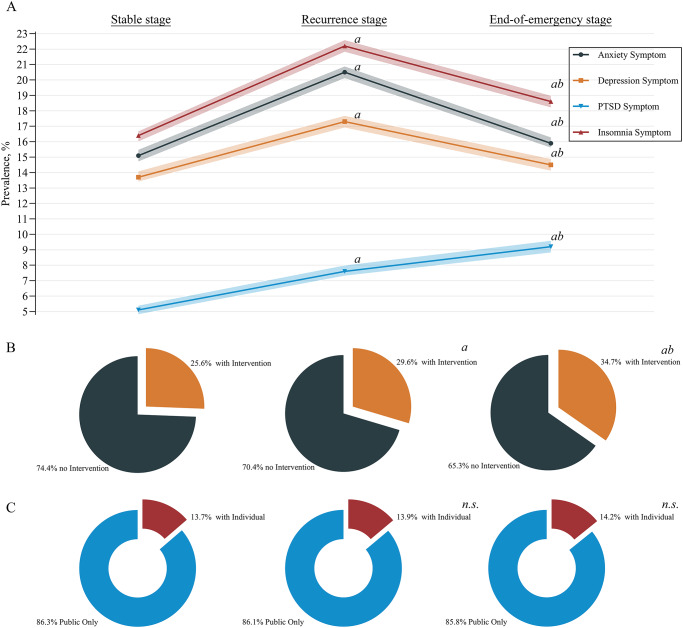
Line chart showing trends in the prevalence of mental health symptoms (**A**) and sector chart showing the proportions of participants who received psychological intervention (**B**) and the distribution of intervention types (**C**; public psychological education only or with individual counselling) during the COVID-19 pandemic. COVID-19, coronavirus disease 2019; PTSD, post-traumatic stress disorder; *n.S.*, Not significant. ^A^(Bonferroni) adjusted *P* < 0.05 compared with the stable stage (post hoc *z*-test for pairwise comparisons, adjusted by Bonferroni correction); ^b^(Bonferroni) adjusted *P* < 0.05 compared with the recurrence stage (post hoc *z*-test for pairwise comparisons, adjusted by Bonferroni correction).

In addition, although a significant increase was observed in participants who accepted psychological intervention (9,266 [25.6%] vs 10,678 [29.6%] vs 12,594 [34.7%]), the proportions of individual counselling did not significantly change for these 3 stages (13.7% [1269] vs 13.9% [1482] vs 14.2% [1,791]) (Fig. 2).

### Prevalence of mental health symptoms at different pandemic stages

The prevalence of mental health symptoms at different pandemic stages is shown in Table 2 and Fig. 2. The prevalence of anxiety symptoms increased from 15.1% (95% CI, 14.7–15.5%) at the stable stage to 20.5% (20.1–20.9%) at the recurrence stage and decreased to 15.9% (15.6–16.3%) at the end-of-emergency stage. Similar trends were also revealed in the prevalences of depression (13.7% [13.4–14.1%] vs 17.3% [16.9–17.7%] vs 14.5% [14.1–14.9%]) and insomnia (16.4% [16.0–16.7%] vs 22.2% [21.8–22.6%] vs 18.6% [18.2–19.0%]) symptoms. In addition, severe symptoms were observed as anxiety in 1.6% (1.4–1.7%), 3.3% (3.1–3.4%), and 2.2% (2.0–2.3%) of participants; depression in 1.2% (1.1–1.3%), 2.4% (2.2–2.5%), and 2.0% (1.9–2.1%) of participants; and insomnia in 1.7% (1.6–1.8%), 4.1% (3.9–4.3%) and 3.0% (2.8–3.1%) of participants at the stable, recurrence and end-of-emergency stages, respectively, similar to the aforementioned trends. Although symptoms and severe symptoms decreased from the recurrence stage to the end-of-emergency stage, their prevalence was still greater than that at the stable stage. However, the prevalence of PTSD symptoms continuously increased from 5.1% (4.9–5.3%) at the stable stage to 7.6% (7.4–7.9%) at the recurrence stage and reached 9.2% (8.9–9.5%) at the end-of-emergency stage. Severe PTSD symptoms increased from 0.8% (0.7–0.9%) to 1.4% (1.3–1.5%) to 1.9% (1.8–2.1%).

**Table 2.** Prevalence of mental health symptoms of all included participants at different COVID-19 pandemic stages (*n*_Stable_ = 36,218, *n*_Recurrence_ = 36,097, and *n*_End-of-emergency_ = 36,306)

	No. % (95% CI)			
Subject	Stable stage (sampled 2021)	Recurrence stage (sampled 2022)	End-of-emergency stage (sampled 2023)	*P* value
Assessment scale/mental health symptom				
GAD-7				
Anxiety symptom (scores ≥10)	5,467	7,410^a^	5,788^a,b^	<0.001*
Prevalence, % (95% CI)	15.1 (14.7−15.5)	20.5 (20.1−20.9)	15.9 (15.6−16.3)	
Severe anxiety symptom (scores ≥15)	567	1,177^a^	784^a,b^	<0.001*
Prevalence, % (95% CI)	1.6 (1.4−1.7)	3.3 (3.1−3.4)	2.2 (2.0−2.3)	
PHQ-9				
Depression symptom (scores ≥10)	4,968	6,258^a^	5,266^a,b^	<0.001*
Prevalence, % (95% CI)	13.7 (13.4−14.1)	17.3 (16.9−17.7)	14.5 (14.1−14.9)	
Severe depression symptom (scores ≥15)	433	862^a^	724^a,b^	<0.001*
Prevalence, % (95% CI)	1.2 (1.1−1.3)	2.4 (2.2−2.5)	2.0 (1.9−2.1)	
IES-R				
PTSD symptom (scores ≥33)	1,853	2,757^a^	3,344^a,b^	<0.001*
Prevalence, % (95% CI)	5.1 (4.9−5.3)	7.6 (7.4−7.9)	9.2 (8.9−9.5)	
Severe PTSD symptom (scores ≥37)	295	509^a^	697^a,b^	<0.001*
Prevalence, % (95% CI)	0.8 (0.7−0.9)	1.4 (1.3−1.5)	1.9 (1.8−2.1)	
ISI				
Insomnia symptom (scores ≥15)	5,925	8,007^a^	6,768^a,b^	<0.001*
Prevalence, % (95% CI)	16.4 (16.0−16.7)	22.2 (21.8−22.6)	18.6 (18.2−19.0)	
Severe insomnia symptom (scores ≥22)	615	1,471^a^	1,074^a,b^	<0.001*
Prevalence, % (95% CI)	1.7 (1.6−1.8)	4.1 (3.9−4.3)	3.0 (2.8−3.1)	

COVID-19, coronavirus disease 2019; GAD-7, Generalized Anxiety Disorder-7 scale; PHQ-9, Patient Health Questionnaire-9; IES-R, Impact of Events Scale-Revised; PTSD, post-traumatic stress disorder; ISI, Insomnia Severity Index; CI, confidence interval. *P < 0.01 (Pearson’s *χ^2^* test).

a (Bonferroni) adjusted *P* < 0.05 comparing with the Stable stage (post hoc *z*-test for pairwise comparisons, adjusted by Bonferroni correction). ^b^(Bonferroni) adjusted *P* < 0.05 comparing with the Recurrence stage (post hoc *z*-test for pairwise comparisons, adjusted by Bonferroni correction).

### Factors associated with mental health symptoms

**Fig. 3.**
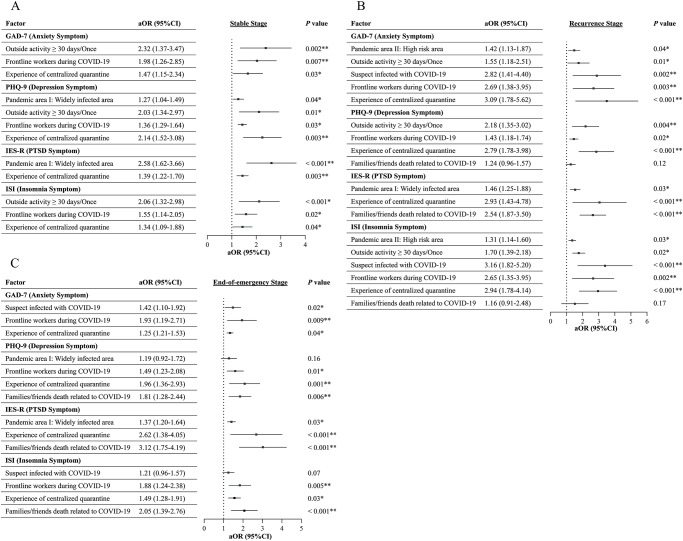
Factors associated with mental health at stable (A), recurrence (B) and end-of-emergency (C) COVID-19 pandemic stages. COVID-19, coronavirus disease 2019; GAD-7, Generalized Anxiety Disorder-7 scale; PHQ-9, Patient Health Questionnaire-9; IES-R, Impact of Events Scale-Revised; PTSD, post-traumatic stress disorder; ISI, Insomnia Severity Index; CI, confidence interval. The factors with significance in the univariable analyses (refer to **Supplementary Tables 2–5**) were then entered into the multivariable logistic regression in a backward fashion to adjust for confounding effects of other factors included in the model. The contrast was set as an indicator determined by the group with the lowest prevalence (proportions) of anxiety, depression, PTSD, and insomnia symptoms to identify potential risk factors for mental health symptoms. The multicollinearity diagnostics showed that variables that were included in the multivariable analyses did not have significant multicollinearity (all variance inflation factors, VIF < 10). *P < 0.05 (multivariable logistic regression); **P < 0.01 (multivariable logistic regression).

Unadjusted univariable logistic regressions are presented in **Supplementary Tables 2-5**. After controlling for confounders, multivariable analyses (Table 3 and Fig. 3) revealed that residents of widely infected areas in the initial wave (i.e., Hubei) had a greater risk of symptoms of depression (aOR, 1.27 [95% CI, 1.04–1.49]) and PSTD (aOR, 2.58 [1.62–3.66]) at the stable stage and PTSD at the recurrence stage (aOR, 1.46 [1.25–1.88]) and the end-of-emergency stage (aOR, 1.37 [1.20–1.64]). Living in high-risk areas in the recurrence wave (i.e., Shanghai and Jilin) was associated with a greater risk of symptoms of anxiety (aOR, 1.42 [1.13–1.87]) and insomnia (aOR, 1.31 [1.14–1.60]) at the recurrence stage. Having engaged in outside activities once in ≥30 days had increased odds of having symptoms of anxiety (aOR, 2.32 [1.37–3.47]), depression (aOR, 2.03 [1.34-2.97]) and insomnia (aOR, 2.06 [1.32–2.98]) at the stable stage and anxiety (aOR, 1.55 [1.18–2.51]), depression (aOR, 2.18 [1.35–3.02]) and insomnia (aOR, 1.70 [1.39–2.18]) at the recurrence stage. Participants with suspected infection demonstrated a greater risk of symptoms of anxiety (aOR, 2.82 [1.41–4.40]) and insomnia (aOR, 3.16 [1.82-5.20]) at the recurrence stage and anxiety (aOR, 1.42 [1.10–1.92]) at the end-of-emergency stage. Serving as COVID-19 frontline workers was a common risk factor for symptoms of anxiety (aOR, 1.98 [1.26–2.85]), depression (aOR, 1.36 [1.29–1.64]) and insomnia (aOR, 1.55 [1.14–2.05]) at the stable stage; anxiety (aOR, 2.69 [1.38–3.95]), depression (aOR, 1.43 [1.18–1.74]) and insomnia (aOR, 2.65 [1.35–3.95]) at the recurrence stage; and anxiety (aOR, 1.93 [1.19–2.71]), depression (aOR, 1.49 [1.23–2.08]), and insomnia (aOR, 1.88 [1.24–2.38]) at the end-of-emergency stage. In addition, individuals who experienced centralized quarantine had an elevated risk of symptoms of anxiety (aOR, 1.47 [1.15–2.34]), depression (aOR, 2.14 [1.52–3.08]), PTSD (aOR, 1.39 [1.22–1.70]) and insomnia (aOR, 1.34 [1.09–1.88]) at the stable stage; anxiety (aOR, 3.09 [1.78–5.62]), depression (aOR, 2.79 [1.78–3.98]), PTSD (aOR, 2.93 [1.43–4.78]) and insomnia (aOR, 2.94 [1.78–4.14]) at the recurrence stage; and anxiety (aOR, 1.25 [1.21–1.53]), depression (aOR, 1.96 [1.36–2.93]), PTSD (aOR, 2.62 [1.38–4.05]) and insomnia (aOR, 1.49 [1.28–1.91]) at the end-of-emergency stage. Family/friends’ deaths were also associated with a greater risk of symptoms of PTSD (aOR, 2.54 [1.87–3.50]) at the recurrence stage and depression (aOR, 1.81 [1.28–2.44]), PTSD (aOR, 3.12 [1.75–4.19]) and insomnia (aOR, 2.05 [1.39–2.76]) at the end-of-emergency stage.

**Table 3.** Multivariable logistic regression in identifying independent influential factors of psychological symptoms and interventions of all included participants at different pandemic stages (*n*_Stable_ = 36,218, *n*_Recurrence_ = 36,097 and *n*_End-of-emergency_ = 36,306)

		Stable stage (sampled 2021)		Recurrence stage (sampled 2022)		End-of-emergency stage (sampled 2023)	
Scale /intervention	Factor	aOR (95% CI)	*P* value	aOR (95% CI)	*P* value	aOR (95% CI)	*P* value
GAD-7 (anxiety symptom)	Pandemic area II: High-risk area	NA	NA	1.42 (1.13−1.87)	0.04*	NA	NA
	Outside activity ≥30 days/once	2.32 (1.37−3.47)	0.002**	1.55 (1.18−2.51)	0.01*	NA	NA
	Suspect infected with COVID-19	NA	NA	2.82 (1.41−4.40)	0.002**	1.42 (1.10−1.92)	0.02*
	Frontline workers during COVID-19	1.98 (1.26−2.85)	0.007**	2.69 (1.38−3.95)	0.003**	1.93 (1.19−2.71)	0.009**
	Experience of centralized quarantine	1.47 (1.15−2.34)	0.03*	3.09 (1.78−5.62)	<0.001**	1.25 (1.21−1.53)	0.04*
PHQ-9 (depression symptom)	Pandemic area I: Widely infected area	1.27 (1.04−1.49)	0.04*	NA	NA	1.19 (0.92−1.72)	0.16
	Outside activity ≥30 days/once	2.03 (1.34−2.97)	0.01*	2.18 (1.35−3.02)	0.004**	NA	NA
	Frontline workers during COVID -19	1.36 (1.29−1.64)	0.03*	1.43 (1.18−1.74)	0.02*	1.49 (1.23−2.08)	0.01*
	Experience of centralized quarantine	2.14 (1.52−3.08)	0.003**	2.79 (1.78−3.98)	<0.001**	1.96 (1.36−2.93)	0.001**
	Families/friends death related to COVID-19	NA	NA	1.24 (0.96−1.57)	0.12	1.81 (1.28− 2.44)	0.006**
IES-R (PTSD symptom)	Pandemic area I: Widely infected area	2.58 (1.62− 3.66)	<0.001**	1.46 (1.25 − 1.88)	0.03*	1.37 (1.20−1.64)	0.03*
	Experience of centralized quarantine	1.39 (1.22−1.70)	0.003**	2.93 (1.43−4.78)	<0.001**	2.62 (1.38−4.05)	<0.001**
	Families/friends death related to COVID-19	NA	NA	2.54 (1.87−3.50)	<0.001**	3.12 (1.75−4.19)	<0.001**
ISI (insomnia symptom)	Pandemic area II: High-risk area	NA	NA	1.31 (1.14−1.60)	0.03*	NA	NA
	Outside activity ≥30 days/Once	2.06 (1.32−2.98)	<0.001*	1.70 (1.39−2.18)	0.02*	NA	NA
	Suspect infected with COVID-19	NA	NA	3.16 (1.82−5.20)	<0.001**	1.21 (0.96−1.57)	0.07
	Frontline workers during COVID-19	1.55 (1.14−2.05)	0.02*	2.65 (1.35−3.95)	0.002**	1.88 (1.24−2.38)	0.005**
	Experience of centralized quarantine	1.34 (1.09−1.88)	0.04*	2.94 (1.78−4.14)	<0.001**	1.49 (1.28−1.91)	0.03*
	Families/friends death related to COVID-19	NA	NA	1.16 (0.91−2.48)	0.17	2.05 (1.39−2.76)	<0.001**

The factors with significance in the univariable analyses (refer to **Supplementary Table 2–5**) were then entered into the multivariable logistic regression in a backward fashion to adjust for confounding effects of other factors included in the model. The contrast was set as an indicator determined by the group with lowest prevalences (proportions) of anxiety symptoms, depression symptoms, insomnia symptoms and psychological interventions to identify risk factors. The multicollinearity diagnostics showed variables that were included in the multivariable analyses did not have significant multicollinearity (all variance inflation factors, VIF < 10). COVID-19, coronavirus disease 2019; GAD-7, Generalized Anxiety Disorder-7 scale; PHQ-9, Patient Health Questionnaire-9; IES-R, Impact of Events Scale-Revised; ISI, Insomnia Severity Index; PTSD, post-traumatic stress disorder; NA, not applicable.

* *P* < 0.05 (multivariable logistic regression); **P < 0.01 (multivariable logistic regression).

## Discussion

Up to date, this is the largest nationwide repeated cross-sectional study on mental health symptoms and associated factors at different COVID-19 pandemic periods for general population, of which dataset can have important contributions to the global scientific community. In total, 36,218, 36,097 and 36,306 participants with sufficient national representativeness were enrolled at 3 crucial stages: stable, recurrence and end of emergency, respectively. The prevalence of anxiety, depression and insomnia symptoms exhibited a similar trend, increasing from 13.7–16.4% at the stable stage to 17.3–22.2% at the recurrence stage. Although the prevalence decreased to 14.5–18.6% at the end-of-emergency stage, it was still higher than that at the stable stage. The prevalence of PTSD symptoms continuously increased from 5.1% at the stable stage to 7.6% and 9.2% at the recurrence and end-of-emergency stages, respectively. Several factors were also associated with a greater risk of mental symptoms, including centralized quarantine, frontline workers, and initial wave widely infected area at all 3 stages; lack of outside activity at the stable and recurrence stages; high-risk areas at the recurrence stage; and suspected infection and family/friends’ deaths at the recurrence and end-of-emergency stages. These findings could more reliably inform public health policies and population-specific strategies and could be an important reference for future potential pandemics and recurrence (Aknin *et al.*, 2022).

Estimates of mental health status are closely related to the measurement scales and their cut-off thresholds (Salanti *et al.*, 2022; Salari *et al.*, 2020; Xiong *et al.*, 2020). In the present study, we applied the cut-offs that optimally prompt probable clinical diagnoses of anxiety, depression, PTSD and insomnia as scores above moderate symptoms (the best cut-off for a probable diagnosis) to avoid overestimation (mild preclinical symptoms) or underestimation (severe symptoms) (Chung *et al.*, 2011; Creamer *et al.*, 2003; Thorndike *et al.*, 2011; Wang *et al.*, 2014; Wu and Chan, 2003; Zhang *et al.*, 2013, 2021). In addition, this stratification also improved the compatibility of the current findings with those of pre-COVID-19 studies on mental disorders (Huang *et al.*, 2019; Lu *et al.*, 2021), other COVID-19 studies (Chen *et al.*, 2023; Salanti *et al.*, 2022; Salari *et al.*, 2020; Xiong *et al.*, 2020), and our previous estimates(Wang *et al.*, 2020, 2021; Zhang *et al.*, 2020). However, it should also be noted that the current samples are all from Chinese populations, and the scales and cut-off values are based on Chinese norms. Although theoretically, the severity classification standards represented by these cut-off values are consistent with those of international samples, further research is needed to validate their international applicability and comparability. Generally, compared with the pre-COVID-19 prevalence of mental disorders (3.6–5.0% for anxiety, depression and other disorders; and 15.0% for insomnia) in the general population in China (Cao *et al.*, 2017; Huang *et al.*, 2019; Kola *et al.*, 2021), there is a clear indication of an upward burden of mental health problems across different pandemic periods, even after the end of emergency.

During the COVID-19 outbreak, an overall 11.0–31.9% prevalence of mental health symptoms was reported (Chen *et al.*, 2023; Ettman *et al.*, 2020; Salanti *et al.*, 2022; Salari *et al.*, 2020; Xiong *et al.*, 2020). A great number of national and regional governments have applied drastic outside activity restrictions and strict interpersonal isolation, while panic (Hossain *et al.*, 2020; Xiong *et al.*, 2020), quarantine (Jin *et al.*, 2021; Kelly, 2021), hospitalization (Patel *et al.*, 2022), physical distancing (Shi *et al.*, 2020; Wang *et al.*, 2020) and policy stringency (Aknin *et al.*, 2022) have contributed to serious psychiatric epidemics co-occurring with COVID-19(Hossain *et al.*, 2020). After the initial waves of the pandemic, changes in mental health symptoms varied substantially across studies (Patel *et al.*, 2022; Salanti *et al.*, 2022). For the Chinese government, work resumption and the ‘normalized prevention and control’ policy were announced in February 2020. A slight increase in the prevalence of anxiety, depression, and insomnia symptoms was observed at 14.9–18.3% (versus 11.0–13.3% with a similar design for the initial wave) at the beginning of work resumption (Wang *et al.*, 2021; Zhang *et al.*, 2020)^,^ and a lower prevalence of symptoms (10.8–16.4%) was reported in later surveys and this study (Tan *et al.*, 2020), as the pandemic was gradually controlled with vaccines(Fiolet *et al.*, 2022; Polack *et al.*, 2020). A time series analysis of electronic healthcare records also suggested reductions in primary care-recorded self-harm following the onset of the pandemic (Pirkis *et al.*, 2021). The present study captured a crucial pandemic recurrence period when new virus mutations occurred and caused recurrent infections(El-Shabasy *et al.*, 2022; Yisimayi *et al.*, 2024), during which the Chinese government announced the ‘dynamic zero’ policy (Bai *et al.*, 2022), a strategy similar to the test-trace-quarantine strategy but still widely restricted cross-regional activities(Kerr *et al.*, 2021). There was a significant increase in mental health symptoms (17.3–22.2%) at the recurrence stage, suggesting the synchronous nature of the mental health crisis. In December 2022, the Chinese national government announced the end-of-emergency of the pandemic and abolished restrictions. Our survey at 6 months after this time point suggested that mental health symptoms decreased but remained in 14.5–18.6% of the population, suggesting potential time lag effects and post-acute symptoms (Penninx *et al.*, 2022). Experience from other epidemics, such as severe acute respiratory syndrome (SARS), suggested long-term mental health consequences, which could last for more than 3 years (Liu *et al.*, 2012; Wu *et al.*, 2009). The impacts of chronic mental health necessitate the attention of the global health community and require future studies. The continuous increase in PTSD symptoms is noteworthy. During the recurrence and end-of-emergency periods, the rapid spread of the virus and insufficient medical resources have led to more individuals experiencing quarantine and deaths from family/friends, which could result in traumatic experiences and cause PTSD symptoms (Cao *et al.*, 2022; Cénat *et al.*, 2021; Chamaa *et al.*, 2021; Chen *et al.*, 2023; Dubey *et al.*, 2020; Jafri *et al.*, 2022), requiring timely preventive and treatment measures. Together, our findings systematically traced the temporalities of the mental health impacts of different COVID-19 pandemic periods and highlighted the increases in mental health symptoms when the pandemic recurred and symptoms remained even after the end of the emergency, especially PTSD symptoms.

Centralized quarantine, frontline workers, and initial wave widely infected area were persistent risk factors for mental health symptoms at all 3 stages. Under centralized quarantine, people might experience multiple pressures, such as infection concerns and interpersonal isolation(Jin *et al.*, 2021; Kelly, 2021; Shi *et al.*, 2020). An epidemiological study of the general population in China during the COVID-19 outbreak also revealed that quarantine was associated with depression, anxiety, insomnia, and acute stress symptoms(Shi *et al.*, 2020), while the current study further confirmed the long-term existence of this risk factor. They also reported that home quarantine contributed to poor mental health (Shi *et al.*, 2020), which was not observed in this study. We assume that this discrepancy is related to expanded knowledge of the virus after the initial waves and more easily accessed psychosocial support at home (Parotto *et al.*, 2023). In addition, this study, together with previous studies, suggested long-lasting negative effects, such as PTSD symptoms, requiring sufficient management (Brooks *et al.*, 2020; Dubey *et al.*, 2020). Frontline workers also reported sustained symptoms of anxiety, depression and insomnia, which was also suggested in studies during the outbreak (Dubey *et al.*, 2020; Lai *et al.*, 2020; Wang *et al.*, 2020, 2021). Frontline workers experienced greater occupational exposure risks, increased work overtime and nightshifts, and more frequently witnessed suffering and death of infected patients, requiring particular attention to their mental health and fostering a resilient work environment (Berkhout *et al.*, 2021; Labrague, 2021; Lai *et al.*, 2020). Another prominent finding was that residents of the initially wide infected area (i.e., Hubei) had a persistently greater risk of PTSD symptoms. Populations in Hubei (including Wuhan city), which experienced early transmission of COVID-19 in China, experienced the most worries and uncertainty about the pandemic and the initial strict outside activity restrictions and had the highest rates of infection and deaths during the outbreak (Huang *et al.*, 2020; Zhu *et al.*, 2020). In addition to many studies during the initial waves reporting a high risk of psychological distress in Hubei (Lai *et al.*, 2020; Shi *et al.*, 2020; Wang *et al.*, 2020, 2021), this study also revealed post-acute symptoms even after the end of emergency, indicating that persistent symptoms are still challenging for residents in this area.

Some stage-specific risk factors were also identified. A lack of outside activity was identified as a risk factor for anxiety, depression and insomnia symptoms during the previous outbreak and return-to-work periods of COVID-19 (Creese *et al.*, 2021; Wang *et al.*, 2020, 2021; Zhang *et al.*, 2020), while current findings suggest that engaging in outside activity once in ≥ 30 days also increases the risk of anxiety, depression and insomnia symptoms at the stable and recurrence stages. As restrictions and isolation controls were abolished after the end of the emergency, the frequency of outside activities was quickly restored, and it no longer served as risk factors. At the recurrence stage, living in a high-risk area was also associated with increased odds of anxiety and insomnia symptoms, suggesting the rationality of mediating mental health policy when a pandemic occurs. With growing populations and their families or friends having experienced the infection, participants with suspected infections and who experienced family/friends’ deaths were at a greater risk of symptoms at the recurrence and end-of-emergency stages. These negative experiences and events were also found to have impacts on mental health during the outbreak (Hossain *et al.*, 2020; Shi *et al.*, 2020; Wang *et al.*, 2020, 2021), inspiring us to protect vulnerable individuals in a timely manner(Penninx *et al.*, 2022). These identified risk factors could contribute to the delivery of effective targeted interventions for those at risk for mental health symptoms(Penninx *et al.*, 2022; Shi *et al.*, 2020; Wang *et al.*, 2020). In addition, demographic factors such as sex and age were also previously reported but with substantial heterogeneity and controversial results (Lai *et al.*, 2020; Penninx *et al.*, 2022; Shi *et al.*, 2020), while the findings of the current study were not significant. This variation might be related to the sample, location and culture, and future controlled studies are needed.

Finally, although a significant increase in the proportion of patients receiving psychological intervention was observed, only limited of them have received individual counselling, which were still not enough to cover potential mental health burdens (Hossain *et al.*, 2020; Kola *et al.*, 2021; Salari *et al.*, 2020). Public psychological education has its advantages as providing some knowledge in relieving psychological distress and promoting those with potential symptoms to pursue professional interventions. But for people who have already developed mental health symptoms, especially severe symptoms, public education is not enough, while individual counselling provided by professional psychologists with systematic terms and individualized treatments could be more effectively (Hossain *et al.*, 2020; Labrague, 2021; Wang *et al.*, 2021). While pandemic during an infectious could be a non-negligible barrier for face-to-face psychological interventions, an important lesson was the growing evidence of remotely computerized or videoconferencing delivered interventions during COVID-19, which guides more safety measures (Bryant *et al.*, 2022; Liu *et al.*, 2021). However, their efficacy, stability, acceptability and applicability still need future comparisons with traditional well developed measures. To better address changes in the mental health burdens of society, psychosocial crisis prevention and multipronged intervention models should be urgently developed at the level of individualization by the government, healthcare providers, and other stakeholders in preparation for future potential pandemics and recurrences (Dubey *et al.*, 2020; Dzinamarira *et al.*, 2024). Furthermore, considering potential post-acute and long-term symptoms, prolonged evidence-based interventions should be applied to address mental health and unfavourable socio-environmental factors for at-risk populations (Ettman *et al.*, 2020; Penninx *et al.*, 2022).

Our study has some limitations. First, because of pandemic restrictions, random sampling was unavailable, which might introduce selection bias. Second, due to the nature of cross-sectional surveys, the present study did not follow the same group due to sensitivity concerns. Additionally, the participants in the current study were surveyed only 6 months after the end of emergency. A longitudinal cohort study with longer follow-ups would be better for exploring changes in and long-term effects of mental health symptoms. Third, comparisons of the current findings with those of other studies could reveal heterogeneity in sample and methodology (especially for the assessment scales used and their cut-offs), and findings from the current study still need to be verified in other countries/regions and international collaboration, which could be a future research direction. Finally, the measurement of mental health symptoms was based on self-reported screening tools, which cannot represent clinical diagnoses.

## Conclusions

The prevalence of anxiety, depression, and insomnia symptoms increased from 13.7–16.4% at the stable stage of the COVID-19 pandemic to 17.3–22.2% at the recurrence stage. Although the prevalence decreased to 14.5–18.6% at the end-of-emergency stage, it was still higher than that at the stable stage. The prevalence of PTSD symptoms continuously increased from 5.1% at the stable stage to 7.6% and 9.2% at the recurrence and end-of-emergency stages, respectively. Several key factors and their variations were identified at different pandemic stages. Centralized quarantine, frontline workers and initial wave-widely infected areas had a persistent increase in the risk of symptoms, while stage-specific risk factors included a lack of outside activity, high-risk recurrence areas, suspected infections and family/friend deaths, suggesting potential differences in at-risk populations. Current individual counselling still does not cover those potentially experienced mental health symptoms enough.

## Supporting information

10.1017/S2045796025100243.sm001 Wang et al. supplementary material 1 Wang et al. supplementary material

10.1017/S2045796025100243.sm002 Wang et al. supplementary material 2 Wang et al. supplementary material

10.1017/S2045796025100243.sm003 Wang et al. supplementary material 3 Wang et al. supplementary material

10.1017/S2045796025100243.sm004 Wang et al. supplementary material 4 Wang et al. supplementary material

10.1017/S2045796025100243.sm005 Wang et al. supplementary material 5 Wang et al. supplementary material

## Data Availability

The data that support the findings of this study are included in the article/supplementary material. More information is available on request from the corresponding authors upon reasonable request.

## Notes

**List of abbreviations**
GAD-7: Generalized Anxiety Disorder-7 Scale
PHQ-9: Patient Health Questionnaire-9
ISI: Insomnia Severity Index
COVID-19: coronavirus disease 2019
aORs: adjusted odds ratios
PTSD: post-traumatic stress disorder.

## Supplementary material

The supplementary material for this article can be found at https://doi.org/10.1017/S2045796025100243.
