# Supplementary material for: Mental health symptoms and associated factors for general population at the stable, recurrence, and end-of-emergency stages of the COVID-19 pandemic: a repeated national cross-sectional study
Source: Epidemiol Psychiatr Sci. 2025 Oct 14;34:e50. doi: 10.1017/S2045796025100243 (PMC12555081; doi:10.1017/S2045796025100243)
Supplement: Wang et al. supplementary material 1 — Wang et al. supplementary material [file S2045796025100243sup001.docx]

Supplementary Table 1. Region division for provinces at different COVID-19 pandemic stages.

| Subject | Province | No. of participants included | | |
| --- | --- | --- | --- | --- |
|  |  | Stable stage  (Sampled 2021) | Recurrence stage  (Sampled 2022) | End-of-emergency stage  (Sampled 2023) |
| Region division^†^  (No. of participants included) |  |  |  |  |
| Socio-geographic region (NEPD, normal period) | |  |  |  |
| Eastern region  (*n_Stable_* = 14,554, *n_Recurrence_* = 14,583, and *n_End-of-emergency_* = 14,611) | Beijing | 604 | 596 | 617 |
|  | Tianjin | 341 | 350 | 345 |
|  | Hebei | 1,801 | 1,809 | 1,814 |
|  | Shanghai | 688 | 716 | 706 |
|  | Jiangsu | 2,155 | 2,141 | 2,165 |
|  | Zhejiang | 1,802 | 1,814 | 1,808 |
|  | Fujian | 1,039 | 1,034 | 1,038 |
|  | Shandong | 2,591 | 2,599 | 2,591 |
|  | Guangdong | 3,280 | 3,260 | 3,273 |
|  | Hainan | 253 | 264 | 254 |
| Middle region  (*n_Stable_* = 8,976, *n_Recurrence_* = 8,893, and *n_End-of-emergency_* = 8,970) | Shanxi | 953 | 927 | 932 |
|  | Anhui | 1,554 | 1,529 | 1,565 |
|  | Jiangxi | 1,038 | 1,023 | 1,035 |
|  | Henan | 2,323 | 2,325 | 2,330 |
|  | Hubei | 1,463 | 1,465 | 1,471 |
|  | Hunan | 1,645 | 1,624 | 1,637 |
| Western region  (*n_Stable_* = 9,759, *n_Recurrence_* = 9,724, and *n_End-of-emergency_* = 9,716) | Inner Mongolia | 696 | 677 | 688 |
|  | Guangxi | 1,207 | 1,204 | 1,216 |
|  | Chongqing | 870 | 869 | 870 |
|  | Sichuan | 2,236 | 2,225 | 2,239 |
|  | Guizhou | 957 | 942 | 921 |
|  | Yunnan | 1,124 | 1,112 | 1,123 |
|  | Tibet | 82 | 92 | 76 |
|  | Gansu | 618 | 605 | 602 |
|  | Shaanxi | 1,038 | 1,041 | 1,046 |
|  | Qinghai | 168 | 168 | 174 |
|  | Ningxia | 166 | 180 | 179 |
|  | Xinjiang | 597 | 609 | 582 |
| Northeast region  (*n_Stable_* = 2,929, *n_Recurrence_* = 2,897, and *n_End-of-emergency_* = 3,009) | Liaoning | 1,289 | 1,271 | 1,317 |
|  | Jilin | 684 | 679 | 713 |
|  | Heilongjiang | 956 | 947 | 979 |
| COVID-19 pandemic area I (initial wave, 2020) | |  |  |  |
| Widely infected area  (*n_Stable_* = 1,463, *n_Recurrence_* = 1,465, and *n_End-of-emergency_* = 1,471) | Hubei | 1,463 | 1,465 | 1,471 |
| Moderate infected area  (*n_Stable_* = 20,098, *n_Recurrence_* = 20,005, and *n_End-of-emergency_* = 20,130) | Guangdong | 3,280 | 3,260 | 3,273 |
|  | Henan | 2,323 | 2,325 | 2,330 |
|  | Zhejiang | 1,802 | 1,814 | 1,808 |
|  | Hunan | 1,645 | 1,624 | 1,637 |
|  | Anhui | 1,554 | 1,529 | 1,565 |
|  | Jiangxi | 1,038 | 1,023 | 1,035 |
|  | Shandong | 2,591 | 2,599 | 2,591 |
|  | Jiangsu | 2,155 | 2,141 | 2,165 |
|  | Chongqing | 870 | 869 | 870 |
|  | Beijing | 604 | 596 | 617 |
|  | Sichuan | 2,236 | 2,225 | 2,239 |
| Less infected area  (*n_Stable_* = 14,657, *n_Recurrence_* = 14,627, and *n_End-of-emergency_* = 14,705) | Shanghai | 688 | 716 | 706 |
|  | Heilongjiang | 956 | 947 | 979 |
|  | Fujian | 1,039 | 1,034 | 1,038 |
|  | Hebei | 1,801 | 1,809 | 1,814 |
|  | Guangxi | 1,207 | 1,204 | 1,216 |
|  | Shaanxi | 1,038 | 1,041 | 1,046 |
|  | Yunnan | 1,124 | 1,112 | 1,123 |
|  | Tianjin | 341 | 350 | 345 |
|  | Hainan | 253 | 264 | 254 |
|  | Guizhou | 957 | 942 | 921 |
|  | Gansu | 618 | 605 | 602 |
|  | Shanxi | 953 | 927 | 932 |
|  | Liaoning | 1,289 | 1,271 | 1,317 |
|  | Jilin | 684 | 679 | 713 |
|  | Inner Mongolia | 696 | 677 | 688 |
|  | Xinjiang | 597 | 609 | 582 |
|  | Ningxia | 166 | 180 | 179 |
|  | Qinghai | 168 | 168 | 174 |
|  | Tibet | 82 | 92 | 76 |
| COVID-19 pandemic area II (recurrence, 2022) | |  |  |  |
| High risk area  (*n_Recurrence_* = 1,395, and *n_End-of-emergency_* = 1,419) | Shanghai | NA | 716 | 706 |
|  | Jilin | NA | 679 | 713 |
| Moderate risk area  (*n_Recurrence_* = 19,434, and *n_End-of-emergency_* = 19,567) | Guangdong | NA | 3,260 | 3,273 |
|  | Beijing | NA | 596 | 617 |
|  | Fujian | NA | 1,034 | 1,038 |
|  | Shandong | NA | 2,599 | 2,591 |
|  | Heilongjiang | NA | 947 | 979 |
|  | Zhejiang | NA | 1,814 | 1,808 |
|  | Sichuan | NA | 2,225 | 2,239 |
|  | Tianjin | NA | 350 | 345 |
|  | Liaoning | NA | 1,271 | 1,317 |
|  | Hebei | NA | 1,809 | 1,814 |
|  | Guangxi | NA | 1,204 | 1,216 |
|  | Henan | NA | 2,325 | 2,330 |
| Low risk area  (*n_Recurrence_* = 15,268, and *n_End-of-emergency_* = 15,320) | Jiangsu | NA | 2,141 | 2,165 |
|  | Shaanxi | NA | 1,041 | 1,046 |
|  | Jiangxi | NA | 1,023 | 1,035 |
|  | Gansu | NA | 605 | 602 |
|  | Yunnan | NA | 1,112 | 1,123 |
|  | Hunan | NA | 1,624 | 1,637 |
|  | Inner Mongolia | NA | 677 | 688 |
|  | Shanxi | NA | 927 | 932 |
|  | Qinghai | NA | 168 | 174 |
|  | Hainan | NA | 264 | 254 |
|  | Chongqing | NA | 869 | 870 |
|  | Anhui | NA | 1,529 | 1,565 |
|  | Hubei | NA | 1,465 | 1,471 |
|  | Guizhou | NA | 942 | 921 |
|  | Xinjiang | NA | 609 | 582 |
|  | Ningxia | NA | 180 | 179 |
|  | Tibet | NA | 92 | 76 |
| COVID-19 pandemic area III (end-of-emergency, 2023) | |  |  |  |
| Severe affected area  (*n_End-of-emergency_* = 9,273) | Hubei | NA | NA | 1,471 |
|  | Shanghai | NA | NA | 706 |
|  | Guangdong | NA | NA | 3,273 |
|  | Jilin | NA | NA | 713 |
|  | Beijing | NA | NA | 617 |
|  | Sichuan | NA | NA | 2,239 |
|  | Hainan | NA | NA | 254 |
| Moderate affected area  (*n_End-of-emergency_* = 10,814) | Henan | NA | NA | 2,330 |
|  | Chongqing | NA | NA | 870 |
|  | Inner Mongolia | NA | NA | 688 |
|  | Fujian | NA | NA | 1,038 |
|  | Zhejiang | NA | NA | 1,808 |
|  | Shaanxi | NA | NA | 1,046 |
|  | Heilongjiang | NA | NA | 979 |
|  | Yunnan | NA | NA | 1,123 |
|  | Shanxi | NA | NA | 932 |
| Mild affected area  (*n_End-of-emergency_* = 16,219) | Shandong | NA | NA | 2,591 |
|  | Jiangsu | NA | NA | 2,165 |
|  | Liaoning | NA | NA | 1,317 |
|  | Hebei | NA | NA | 1,814 |
|  | Xinjiang | NA | NA | 582 |
|  | Tianjin | NA | NA | 345 |
|  | Hunan | NA | NA | 1,637 |
|  | Guangxi | NA | NA | 1,216 |
|  | Guizhou | NA | NA | 921 |
|  | Anhui | NA | NA | 1,565 |
|  | Gansu | NA | NA | 602 |
|  | Jiangxi | NA | NA | 1,035 |
|  | Tibet | NA | NA | 76 |
|  | Qinghai | NA | NA | 174 |
|  | Ningxia | NA | NA | 179 |

COVID-19, coronavirus disease 2019; NEPD, National Economic Population Division, by the National Bureau of Statistics, China; NA, not applicable. ^†^Region division was classified based on different socio-geographical characteristics or COVID-19 influences in reflecting regional features and infection risks at different pandemic stages; Socio-geographic region was stratified based on NEPD, the National Bureau of Statistics, China, for normal period; COVID-19 pandemic area I was stratified according to cumulative confirmed cases between January 2020 to March 2020 (initial wave, 2020), data from the National Health Commission, China; COVID-19 pandemic area II was stratified according to cumulative confirmed cases between March 2022 to May 2022 (recurrence, 2022), data from the National Health Commission, China; COVID-19 pandemic area III was stratified according to cumulative confirmed cases between January 2020 to December 2022 (end-of-emergency, 2023).
