# Supplementary material for: Mental health symptoms and associated factors for general population at the stable, recurrence, and end-of-emergency stages of the COVID-19 pandemic: a repeated national cross-sectional study
Source: Epidemiol Psychiatr Sci. 2025 Oct 14;34:e50. doi: 10.1017/S2045796025100243 (PMC12555081; doi:10.1017/S2045796025100243)
Supplement: Wang et al. supplementary material 2 — Wang et al. supplementary material [file S2045796025100243sup002.docx]

Supplementary Table 2. Univariable logistic regression in exploring potential independent factors associated with anxiety symptom (measured by GAD-7) of all included participants at different pandemic stages (*n_Stable_* = 36,218, *n_Recurrence_* = 36,097, and *n_End-of-emergency_* = 36,306).

|  | Stable stage  (Sampled 2021) | | | Recurrence stage  (Sampled 2022) | | | End-of-emergency stage  (Sampled 2023) | | |
| --- | --- | --- | --- | --- | --- | --- | --- | --- | --- |
| Factor | Scores ≥ 10 | Scores < 10 | *P* value | Scores ≥ 10 | Scores < 10 | *P* value | Scores ≥ 10 | Scores < 10 | *P* value |
| Region division |  |  |  |  |  |  |  |  |  |
| Socio-geographic region (NEPD, normal period) |  |  | 0.81 |  |  | 0.12 |  |  | 0.42 |
| Eastern region | 2,208 (15.2) | 12,346 (84.8) |  | 3,050 (20.9) | 11,533 (79.1) |  | 2,376 (16.3) | 12,235 (83.7) |  |
| Middle region | 1,373 (15.3) | 7,603 (84.7) |  | 1,843 (20.7) | 7,050 (79.3) |  | 1,435 (16.0) | 7,535 (84.0) |  |
| Western region | 1,446 (14.8) | 8,313 (85.2) |  | 1,915 (19.7) | 7,809 (80.3) |  | 1,504 (15.5) | 8,212 (84.5) |  |
| Northeast region | 440 (15.0) | 2,489 (85.0) |  | 602 (20.8) | 2,295 (79.2) |  | 473 (15.7) | 2,536 (84.3) |  |
| COVID-19 pandemic area I (initial wave, 2020) |  |  | 0.56 |  |  | 0.10 |  |  | 0.25 |
| Widely infected area (≥ 10,000 confirmed cases) | 233 (15.9) | 1,230 (84.1) |  | 306 (20.9) | 1,159 (79.1) |  | 237 (16.1) | 1,234 (83.9) |  |
| Moderate infected area (≥ 500 confirmed cases) | 3,046 (15.2) | 17,052 (84.8) |  | 4,024 (20.1) | 15,981 (79.9) |  | 3,152 (15.7) | 16,978 (84.3) |  |
| Less infected area (< 500 confirmed cases) | 2,188 (14.9) | 12,469 (85.1) |  | 3,080 (21.1) | 11,547 (78.9) |  | 2,399 (16.3) | 12,306 (83.7) |  |
| COVID-19 pandemic area II (recurrence, 2022) |  |  | NA |  |  | 0.005** |  |  | 0.85 |
| High risk area (≥ 10,000 confirmed cases) | NA | NA |  | 334 (23.9) | 1,061 (76.1) |  | 232 (16.3) | 1,187 (83.7) |  |
| Moderate risk area (≥ 500 confirmed cases) | NA | NA |  | 3,971 (20.4) | 15,463 (79.6) |  | 3,129 (16.0) | 16,438 (84.0) |  |
| Low risk area (< 500 confirmed cases) | NA | NA |  | 3,105 (20.3) | 12,163 (79.7) |  | 2,427 (15.8) | 12,893 (84.2) |  |
| COVID-19 pandemic area III (end-of-emergency, 2023) |  |  | NA |  |  | NA |  |  | 0.45 |
| Severe affected area (≥ 10,000 confirmed cases) | NA | NA |  | NA | NA |  | 1,515 (16.3) | 7,758 (83.7) |  |
| Moderate affected area (≥ 5,000 confirmed cases) | NA | NA |  | NA | NA |  | 1,720 (15.9) | 9,094 (84.1) |  |
| Mild affected area (< 5,000 confirmed cases) | NA | NA |  | NA | NA |  | 2,553 (15.7) | 13,666 (84.3) |  |
| Characteristic |  |  |  |  |  |  |  |  |  |
| Gender |  |  | 0.22 |  |  | 0.10 |  |  | 0.20 |
| Male | 2,759 (14.9) | 15,797 (85.1) |  | 3,712 (20.2) | 14,680 (79.8) |  | 2,922 (15.7) | 15,689 (84.3) |  |
| Female | 2,708 (15.3) | 14,954 (84.7) |  | 3,698 (20.9) | 14,007 (79.1) |  | 2,866 (16.2) | 14,829 (83.8) |  |
| Age, years |  |  |  |  |  |  |  |  |  |
| 18-34 | 1,472 (14.8) | 8,468 (85.2) | 0.72 | 1,966 (19.9) | 7,915 (80.1) | 0.10 | 1,553 (15.5) | 8,474 (84.5) | 0.44 |
| 35-49 | 1,581 (15.2) | 8,843 (84.8) |  | 2,143 (20.8) | 8,184 (79.2) |  | 1,665 (16.1) | 8,673 (83.9) |  |
| 50-64 | 1,487 (15.4) | 8,181 (84.6) |  | 2,027 (21.2) | 7,522 (78.8) |  | 1,589 (16.3) | 8,162 (83.7) |  |
| ≥65 | 927 (15.0) | 5,259 (85.0) |  | 1,274 (20.1) | 5,066 (79.9) |  | 981 (15.8) | 5,209 (84.2) |  |
| Place of residence |  |  | 0.56 |  |  | 0.09 |  |  | 0.16 |
| Urban | 2,947 (15.0) | 16,709 (85.0) |  | 3,975 (20.2) | 15,707 (79.8) |  | 3,093 (15.7) | 16,614 (84.3) |  |
| Rural | 2,520 (15.2) | 14,042 (84.8) |  | 3,435 (20.9) | 12,980 (79.1) |  | 2,695 (16.2) | 13,904 (83.8) |  |
| Education level |  |  | 0.49 |  |  | 0.49 |  |  | 0.25 |
| Less than college | 4,257 (15.0) | 24,075 (85.0) |  | 5,782 (20.4) | 22,492 (79.6) |  | 4,530 (15.8) | 24,092 (84.2) |  |
| College degree or higher | 1,210 (15.3) | 6,676 (84.7) |  | 1,628 (20.8) | 6,195 (79.2) |  | 1,258 (16.4) | 6,426 (83.6) |  |
| Marriage status |  |  |  |  |  |  |  |  |  |
| Unmarried | 1,057 (15.2) | 5,903 (84.8) | 0.87 | 1,446 (20.9) | 5,483 (79.1) | 0.34 | 1,121 (16.0) | 5,902 (84.0) | 0.97 |
| Married | 3,971 (15.0) | 22,432 (85.0) |  | 5,372 (20.4) | 21,025 (79.6) |  | 4,202 (15.9) | 22,191 (84.1) |  |
| Divorced/Widowed | 439 (15.4) | 2,416 (84.6) |  | 592 (21.4) | 2,179 (78.6) |  | 465 (16.1) | 2,425 (83.9) |  |
| History of chronic diseases |  |  | 0.36 |  |  | 0.49 |  |  | 0.38 |
| Yes | 519 (15.9) | 2,736 (84.1) |  | 695 (21.3) | 2,563 (78.7) |  | 553 (16.8) | 2,746 (83.2) |  |
| No | 4,781 (15.0) | 27,054 (85.0) |  | 6,497 (20.4) | 25,280 (79.6) |  | 5,058 (15.8) | 26,858 (84.2) |  |
| Unknown | 167 (14.8) | 961 (85.2) |  | 218 (20.5) | 844 (79.5) |  | 177 (16.2) | 914 (83.8) |  |
| History of psychiatric disorders |  |  | 0.41 |  |  | 0.48 |  |  | 0.24 |
| Yes | 72 (17.4) | 341 (82.6) |  | 98 (22.9) | 330 (77.1) |  | 81 (18.9) | 348 (81.1) |  |
| No | 5,228 (15.1) | 29,480 (84.9) |  | 7,095 (20.5) | 27,515 (79.5) |  | 5,517 (15.9) | 29,187 (84.1) |  |
| Unknown | 167 (15.2) | 930 (84.8) |  | 217 (20.5) | 842 (79.5) |  | 190 (16.2) | 983 (83.8) |  |
| Occupation |  |  | 0.83 |  |  | 0.07 |  |  | 0.78 |
| Students, full-time | 243 (14.1) | 1,480 (85.9) |  | 326 (19.4) | 1,355 (80.6) |  | 266 (15.7) | 1,425 (84.3) |  |
| Technicians and associate professionals | 541 (15.0) | 3,059 (85.0) |  | 724 (19.7) | 2,955 (80.3) |  | 584 (16.2) | 3,029 (83.8) |  |
| Government and clerical support workers | 480 (14.9) | 2,749 (85.1) |  | 689 (21.4) | 2,537 (78.6) |  | 522 (16.1) | 2,727 (83.9) |  |
| Social and life service workers | 1,469 (15.4) | 8,047 (84.6) |  | 2,051 (21.5) | 7,483 (78.5) |  | 1,592 (16.4) | 8,092 (83.6) |  |
| Agricultural, forestry and fishery workers | 1,041 (14.9) | 5,965 (85.1) |  | 1,402 (19.8) | 5,669 (80.2) |  | 1,091 (15.4) | 6,006 (84.6) |  |
| Production and manufacture workers | 1,354 (15.1) | 7,635 (84.9) |  | 1,793 (20.2) | 7,091 (79.8) |  | 1,384 (15.7) | 7,409 (84.3) |  |
| Other unclassified occupations | 15 (14.3) | 90 (85.7) |  | 19 (19.6) | 78 (80.4) |  | 16 (15.5) | 87 (84.5) |  |
| Freelance or inoccupation | 324 (15.8) | 1,726 (84.2) |  | 406 (21.1) | 1,519 (78.9) |  | 333 (16.0) | 1,743 (84.0) |  |
| Yearly family income, CNY |  |  | 0.96 |  |  | 0.37 |  |  | 0.97 |
| <40,000 | 1,139 (15.2) | 6,358 (84.8) |  | 1,522 (20.2) | 6,015 (79.8) |  | 1,208 (16.0) | 6,349 (84.0) |  |
| 40,000-99,999 | 3,426 (15.1) | 19,299 (84.9) |  | 4,710 (20.8) | 17,980 (79.2) |  | 3,638 (16.0) | 19,165 (84.0) |  |
| ≥100,000 | 902 (15.0) | 5,094 (85.0) |  | 1,178 (20.1) | 4,692 (79.9) |  | 942 (15.8) | 5,004 (84.2) |  |
| Activity and work/study status |  |  |  |  |  |  |  |  |  |
| Outside activity/Once |  |  | < 0.001** |  |  | < 0.001** |  |  | 0.07 |
| 1-7 days | 2,738 (14.2) | 16,492 (85.8) |  | 1,459 (18.6) | 6,378 (81.4) |  | 3,650 (15.9) | 19,297 (84.1) |  |
| 8-14 days | 1,584 (14.4) | 9,398 (85.6) |  | 2,206 (18.5) | 9,698 (81.5) |  | 1,582 (15.7) | 8,479 (84.3) |  |
| 15-29 days | 589 (15.1) | 3,319 (84.9) |  | 1,730 (19.4) | 7,190 (80.6) |  | 408 (16.1) | 2,124 (83.9) |  |
| ≥30 days | 556 (26.5) | 1,542 (73.5) |  | 2,015 (27.1) | 5,421 (72.9) |  | 148 (19.3) | 618 (80.7) |  |
| Work/Study status |  |  | 0.41 |  |  | 0.06 |  |  | 0.72 |
| On-site work/study | 3,397 (15.2) | 18,994 (84.8) |  | 2,121 (21.2) | 7,880 (78.8) |  | 4,603 (16.0) | 24,244 (84.0) |  |
| Off-site work/study | 1,119 (15.3) | 6,182 (84.7) |  | 3,402 (20.5) | 13,179 (79.5) |  | 752 (15.6) | 4,058 (84.4) |  |
| Not back to work/study | 951 (14.6) | 5,575 (85.4) |  | 1,887 (19.8) | 7,628 (80.2) |  | 433 (16.3) | 2,216 (83.7) |  |
| Experience related to COVID-19 |  |  |  |  |  |  |  |  |  |
| Current COVID-19 identity |  |  | 0.60 |  |  | < 0.001** |  |  | 0.008** |
| Current infected | 63 (15.7) | 339 (84.3) |  | 894 (20.7) | 3,428 (79.3) |  | 365 (16.2) | 1,892 (83.8) |  |
| Previous infected | 508 (15.2) | 2,841 (84.8) |  | 1,249 (19.9) | 5,042 (80.1) |  | 3,916 (15.8) | 20,905 (84.2) |  |
| Suspect infected | 95 (17.1) | 462 (82.9) |  | 1,692 (31.9) | 3,607 (68.1) |  | 506 (18.2) | 2,277 (81.8) |  |
| Not infected | 4,801 (15.0) | 27,109 (85.0) |  | 3,575 (17.7) | 16,610 (82.3) |  | 1,001 (15.5) | 5,444 (84.5) |  |
| Frontline workers during COVID-19 |  |  | < 0.001** |  |  | < 0.001** |  |  | < 0.001** |
| Yes | 1,413 (23.4) | 4,625 (76.6) |  | 1,998 (29.8) | 4,700 (70.2) |  | 1,750 (21.9) | 6,226 (78.1) |  |
| No | 4,054 (13.4) | 26,126 (86.6) |  | 5,412 (18.4) | 23,987 (81.6) |  | 4,038 (14.3) | 24,292 (85.7) |  |
| Experience of hospitalization for COVID-19 |  |  | 0.41 |  |  | 0.07 |  |  | 0.09 |
| Yes | 424 (15.6) | 2,286 (84.4) |  | 973 (21.6) | 3,542 (78.4) |  | 1,353 (16.5) | 6,823 (83.5) |  |
| No | 5,043 (15.1) | 28,465 (84.9) |  | 6,437 (20.4) | 25,145 (79.6) |  | 4,435 (15.8) | 23,695 (84.2) |  |
| Experience of quarantine during COVID-19 |  |  | < 0.001** |  |  | < 0.001** |  |  | < 0.001** |
| Centralized | 702 (18.5) | 3,086 (81.5) |  | 2,398 (34.3) | 4,591 (65.7) |  | 1,748 (17.3) | 8,377 (82.7) |  |
| At home | 931 (15.0) | 5,288 (85.0) |  | 1,819 (17.7) | 8,475 (82.3) |  | 2,642 (15.5) | 14,355 (84.5) |  |
| None | 3,834 (14.6) | 22,377 (85.4) |  | 3,193 (17.0) | 15,621 (83.0) |  | 1,398 (15.2) | 7,786 (84.8) |  |
| Families/friends hospitalization related to COVID-19 |  |  | 0.21 |  |  | 0.11 |  |  | 0.22 |
| Yes | 859 (15.7) | 4,628 (84.3) |  | 1,924 (21.1) | 7,187 (78.9) |  | 2,663 (16.2) | 13,772 (83.8) |  |
| No | 4,608 (15.0) | 26,123 (85.0) |  | 5,486 (20.3) | 21,500 (79.7) |  | 3,125 (15.7) | 16,746 (84.3) |  |
| Families/friends death related to COVID-19 |  |  | 0.45 |  |  | 0.11 |  |  | 0.18 |
| Yes | 134 (16.0) | 703 (84.0) |  | 803 (21.5) | 2,928 (78.5) |  | 886 (16.6) | 4,464 (83.4) |  |
| No | 5,333 (15.1) | 30,048 (84.9) |  | 6,607 (20.4) | 25,759 (79.6) |  | 4,902 (15.8) | 26,054 (84.2) |  |
| Psychological intervention during COVID-19 |  |  |  |  |  |  |  |  |  |
| Psychological intervention during COVID-19 |  |  | 0.47 |  |  | 0.25 |  |  | 0.54 |
| Yes | 1,420 (15.3) | 7,846 (84.7) |  | 2,232 (20.9) | 8,446 (79.1) |  | 2,028 (16.1) | 10,566 (83.9) |  |
| No | 4,047 (15.0) | 22,905 (85.0) |  | 5,178 (20.4) | 20,241 (79.6) |  | 3,760 (15.9) | 19,952 (84.1) |  |

The factors with significance in the univariable analyses were then entered into the multivariable logistic regression (refer to **Figure 3** for final factors included in the multivariable model). COVID-19, coronavirus disease 2019; GAD-7, Generalized Anxiety Disorder-7 scale; NA, not applicable. **P* < 0.05 (Univariable logistic regression); ***P* < 0.01 (Univariable logistic regression).
