# Supplementary material for: Mental health symptoms and associated factors for general population at the stable, recurrence, and end-of-emergency stages of the COVID-19 pandemic: a repeated national cross-sectional study
Source: Epidemiol Psychiatr Sci. 2025 Oct 14;34:e50. doi: 10.1017/S2045796025100243 (PMC12555081; doi:10.1017/S2045796025100243)
Supplement: Wang et al. supplementary material 3 — Wang et al. supplementary material [file S2045796025100243sup003.docx]

Supplementary Table 3. Univariable logistic regression in exploring potential independent factors associated with depression symptom (measured by PHQ-9) of all included participants at different pandemic stages (*n_Stable_* = 36,218, *n_Recurrence_* = 36,097, and *n_End-of-emergency_* = 36,306).

|  | Stable stage  (Sampled 2021) | | | Recurrence stage  (Sampled 2022) | | | End-of-emergency stage  (Sampled 2023) | | |
| --- | --- | --- | --- | --- | --- | --- | --- | --- | --- |
| Factor | Scores ≥ 10 | Scores < 10 | *P* value | Scores ≥ 10 | Scores < 10 | *P* value | Scores ≥ 10 | Scores < 10 | *P* value |
| Region division |  |  |  |  |  |  |  |  |  |
| Socio-geographic region (NEPD, normal period) |  |  | 0.85 |  |  | 0.71 |  |  | 0.26 |
| Eastern region | 1,992 (13.7) | 12,562 (86.3) |  | 2,563 (17.6) | 12,020 (82.4) |  | 2,168 (14.8) | 12,443 (85.2) |  |
| Middle region | 1,255 (14.0) | 7,721 (86.0) |  | 1,537 (17.3) | 7,356 (82.7) |  | 1,313 (14.6) | 7,657 (85.4) |  |
| Western region | 1,322 (13.5) | 8,437 (86.5) |  | 1,653 (17.0) | 8,071 (83.0) |  | 1,355 (13.9) | 8,361 (86.1) |  |
| Northeast region | 399 (13.6) | 2,530 (86.4) |  | 505 (17.4) | 2,392 (82.6) |  | 430 (14.3) | 2,579 (85.7) |  |
| COVID-19 pandemic area I (initial wave, 2020) |  |  | 0.02* |  |  | 0.80 |  |  | 0.04* |
| Widely infected area (≥ 10,000 confirmed cases) | 235 (16.1) | 1,228 (83.9) |  | 263 (18.0) | 1,202 (82.0) |  | 246 (16.7) | 1,225 (83.3) |  |
| Moderate infected area (≥ 500 confirmed cases) | 2,769 (13.8) | 17,329 (86.2) |  | 3,471 (17.4) | 16,534 (82.6) |  | 2,922 (14.5) | 17,208 (85.5) |  |
| Less infected area (< 500 confirmed cases) | 1,964 (13.4) | 12,693 (86.6) |  | 2,524 (17.3) | 12,103 (82.7) |  | 2,098 (14.3) | 12,607 (85.7) |  |
| COVID-19 pandemic area II (recurrence, 2022) |  |  | NA |  |  | 0.42 |  |  | 0.53 |
| High risk area (≥ 10,000 confirmed cases) | NA | NA |  | 258 (18.5) | 1,137 (81.5) |  | 217 (15.3) | 1,202 (84.7) |  |
| Moderate risk area (≥ 500 confirmed cases) | NA | NA |  | 3,382 (17.4) | 16,052 (82.6) |  | 2,856 (14.6) | 16,711 (85.4) |  |
| Low risk area (< 500 confirmed cases) | NA | NA |  | 2,618 (17.1) | 12,650 (82.9) |  | 2,193 (14.3) | 13,127 (85.7) |  |
| COVID-19 pandemic area III (end-of-emergency, 2023) |  |  | NA |  |  | NA |  |  | 0.10 |
| Severe affected area (≥ 10,000 confirmed cases) | NA | NA |  | NA | NA |  | 1,406 (15.2) | 7,867 (84.8) |  |
| Moderate affected area (≥ 5,000 confirmed cases) | NA | NA |  | NA | NA |  | 1,558 (14.4) | 9,256 (85.6) |  |
| Mild affected area (< 5,000 confirmed cases) | NA | NA |  | NA | NA |  | 2,302 (14.2) | 13,917 (85.8) |  |
| Characteristic |  |  |  |  |  |  |  |  |  |
| Gender |  |  | 0.48 |  |  | 0.15 |  |  | 0.40 |
| Male | 2,522 (13.6) | 16,034 (86.4) |  | 3,137 (17.1) | 15,255 (82.9) |  | 2,671 (14.4) | 15,940 (85.6) |  |
| Female | 2,446 (13.8) | 15,216 (86.2) |  | 3,121 (17.6) | 14,584 (82.4) |  | 2,595 (14.7) | 15,100 (85.3) |  |
| Age, years |  |  | 0.60 |  |  | 0.45 |  |  | 0.73 |
| 18-34 | 1,329 (13.4) | 8,611 (86.6) |  | 1,670 (16.9) | 8,211 (83.1) |  | 1,427 (14.2) | 8,600 (85.8) |  |
| 35-49 | 1,425 (13.7) | 8,999 (86.3) |  | 1,782 (17.3) | 8,545 (82.7) |  | 1,509 (14.6) | 8,829 (85.4) |  |
| 50-64 | 1,352 (14.0) | 8,316 (86.0) |  | 1,694 (17.7) | 7,855 (82.3) |  | 1,440 (14.8) | 8,311 (85.2) |  |
| ≥65 | 862 (13.9) | 5,324 (86.1) |  | 1,112 (17.5) | 5,228 (82.5) |  | 890 (14.4) | 5,300 (85.6) |  |
| Place of residence |  |  | 0.66 |  |  | 0.22 |  |  | 0.41 |
| Urban | 2,682 (13.6) | 16,974 (86.4) |  | 3,368 (17.1) | 16,314 (82.9) |  | 2,831 (14.4) | 16,876 (85.6) |  |
| Rural | 2,286 (13.8) | 14,276 (86.2) |  | 2,890 (17.6) | 13,525 (82.4) |  | 2,435 (14.7) | 14,164 (85.3) |  |
| Education level |  |  | 0.45 |  |  | 0.08 |  |  | 0.24 |
| Less than college | 3,866 (13.6) | 24,466 (86.4) |  | 4,849 (17.2) | 23,425 (82.8) |  | 4,119 (14.4) | 24,503 (85.6) |  |
| College degree or higher | 1,102 (14.0) | 6,784 (86.0) |  | 1,409 (18.0) | 6,414 (82.0) |  | 1,147 (14.9) | 6,537 (85.1) |  |
| Marriage status |  |  | 0.58 |  |  | 0.41 |  |  | 0.67 |
| Unmarried | 972 (14.0) | 5,988 (86.0) |  | 1,217 (17.6) | 5,712 (82.4) |  | 1,025 (14.6) | 5,998 (85.4) |  |
| Married | 3,592 (13.6) | 22,811 (86.4) |  | 4,539 (17.2) | 21,858 (82.8) |  | 3,807 (14.4) | 22,586 (85.6) |  |
| Divorced/Widowed | 404 (14.2) | 2,451 (85.8) |  | 502 (18.1) | 2,269 (81.9) |  | 434 (15.0) | 2,456 (85.0) |  |
| History of chronic diseases |  |  | 0.35 |  |  | 0.39 |  |  | 0.44 |
| Yes | 473 (14.5) | 2,782 (85.5) |  | 593 (18.2) | 2,665 (81.8) |  | 503 (15.2) | 2,796 (84.8) |  |
| No | 4,338 (13.6) | 27,497 (86.4) |  | 5,482 (17.3) | 26,295 (82.7) |  | 4,608 (14.4) | 27,308 (85.6) |  |
| Unknown | 157 (13.9) | 971 (86.1) |  | 183 (17.2) | 879 (82.8) |  | 155 (14.2) | 936 (85.8) |  |
| History of psychiatric disorders |  |  | 0.48 |  |  | 0.16 |  |  | 0.72 |
| Yes | 65 (15.7) | 348 (84.3) |  | 89 (20.8) | 339 (79.2) |  | 68 (15.9) | 361 (84.1) |  |
| No | 4,751 (13.7) | 29,957 (86.3) |  | 5,983 (17.3) | 28,627 (82.7) |  | 5,026 (14.5) | 29,678 (85.5) |  |
| Unknown | 152 (13.9) | 945 (86.1) |  | 186 (17.6) | 873 (82.4) |  | 172 (14.7) | 1,001 (85.3) |  |
| Occupation |  |  | 0.76 |  |  | 0.61 |  |  | 0.76 |
| Students, full-time | 218 (12.7) | 1,505 (87.3) |  | 273 (16.2) | 1,408 (83.8) |  | 234 (13.8) | 1,457 (86.2) |  |
| Technicians and associate professionals | 487 (13.5) | 3,113 (86.5) |  | 622 (16.9) | 3,057 (83.1) |  | 509 (14.1) | 3,104 (85.9) |  |
| Government and clerical support workers | 438 (13.6) | 2,791 (86.4) |  | 571 (17.7) | 2,655 (82.3) |  | 474 (14.6) | 2,775 (85.4) |  |
| Social and life service workers | 1,349 (14.2) | 8,167 (85.8) |  | 1,709 (17.9) | 7,825 (82.1) |  | 1,458 (15.1) | 8,226 (84.9) |  |
| Agricultural, forestry and fishery workers | 939 (13.4) | 6,067 (86.6) |  | 1,198 (16.9) | 5,873 (83.1) |  | 1,008 (14.2) | 6,089 (85.8) |  |
| Production and manufacture workers | 1,239 (13.8) | 7,750 (86.2) |  | 1,531 (17.2) | 7,353 (82.8) |  | 1,264 (14.4) | 7,529 (85.6) |  |
| Other unclassified occupations | 14 (13.3) | 91 (86.7) |  | 16 (16.5) | 81 (83.5) |  | 15 (14.6) | 88 (85.4) |  |
| Freelance or inoccupation | 284 (13.9) | 1,766 (86.1) |  | 338 (17.6) | 1,587 (82.4) |  | 304 (14.6) | 1,772 (85.4) |  |
| Yearly family income, CNY |  |  | 0.81 |  |  | 0.72 |  |  | 0.88 |
| <40,000 | 1,042 (13.9) | 6,455 (86.1) |  | 1,305 (17.3) | 6,232 (82.7) |  | 1,098 (14.5) | 6,459 (85.5) |  |
| 40,000-99,999 | 3,116 (13.7) | 19,609 (86.3) |  | 3,956 (17.4) | 18,734 (82.6) |  | 3,318 (14.6) | 19,485 (85.4) |  |
| ≥100,000 | 810 (13.5) | 5,186 (86.5) |  | 997 (17.0) | 4,873 (83.0) |  | 850 (14.3) | 5,096 (85.7) |  |
| Activity and work/study status |  |  |  |  |  |  |  |  |  |
| Outside activity/Once |  |  | < 0.001** |  |  | < 0.001** |  |  | 0.10 |
| 1-7 days | 2,541 (13.2) | 16,689 (86.8) |  | 1,265 (16.1) | 6,572 (83.9) |  | 3,321 (14.5) | 19,626 (85.5) |  |
| 8-14 days | 1,469 (13.4) | 9,513 (86.6) |  | 1,898 (15.9) | 10,006 (84.1) |  | 1,426 (14.2) | 8,635 (85.8) |  |
| 15-29 days | 537 (13.7) | 3,371 (86.3) |  | 1,503 (16.8) | 7,417 (83.2) |  | 389 (15.4) | 2,143 (84.6) |  |
| ≥30 days | 421 (20.1) | 1,677 (79.9) |  | 1,592 (21.4) | 5,844 (78.6) |  | 130 (17.0) | 636 (83.0) |  |
| Work/Study status |  |  | 0.78 |  |  | 0.89 |  |  | 0.83 |
| On-site work/study | 3,089 (13.8) | 19,302 (86.2) |  | 1,720 (17.2) | 8,281 (82.8) |  | 4,199 (14.6) | 24,648 (85.4) |  |
| Off-site work/study | 1,001 (13.7) | 6,300 (86.3) |  | 2,877 (17.4) | 13,704 (82.6) |  | 684 (14.2) | 4,126 (85.8) |  |
| Not back to work/study | 878 (13.5) | 5,648 (86.5) |  | 1,661 (17.5) | 7,854 (82.5) |  | 383 (14.5) | 2,266 (85.5) |  |
| Experience related to COVID-19 |  |  |  |  |  |  |  |  |  |
| Current COVID-19 identity |  |  | 0.79 |  |  | 0.51 |  |  | 0.94 |
| Current infected | 57 (14.2) | 345 (85.8) |  | 759 (17.6) | 3,563 (82.4) |  | 325 (14.4) | 1,932 (85.6) |  |
| Previous infected | 464 (13.9) | 2,885 (86.1) |  | 1,093 (17.4) | 5,198 (82.6) |  | 3,596 (14.5) | 21,225 (85.5) |  |
| Suspect infected | 84 (15.1) | 473 (84.9) |  | 952 (18.0) | 4,347 (82.0) |  | 415 (14.9) | 2,368 (85.1) |  |
| Not infected | 4,363 (13.7) | 27,547 (86.3) |  | 3,454 (17.1) | 16,731 (82.9) |  | 930 (14.4) | 5,515 (85.6) |  |
| Frontline workers during COVID-19 |  |  | 0.008** |  |  | 0.002** |  |  | < 0.001** |
| Yes | 893 (14.8) | 5,145 (85.2) |  | 1,249 (18.6) | 5,449 (81.4) |  | 1,270 (15.9) | 6,706 (84.1) |  |
| No | 4,075 (13.5) | 26,105 (86.5) |  | 5,009 (17.0) | 24,390 (83.0) |  | 3,996 (14.1) | 24,334 (85.9) |  |
| Experience of hospitalization for COVID-19 |  |  | 0.44 |  |  | 0.19 |  |  | 0.39 |
| Yes | 385 (14.2) | 2,325 (85.8) |  | 814 (18.0) | 3,701 (82.0) |  | 1,210 (14.8) | 6,966 (85.2) |  |
| No | 4,583 (13.7) | 28,925 (86.3) |  | 5,444 (17.2) | 26,138 (82.8) |  | 4,056 (14.4) | 24,074 (85.6) |  |
| Experience of quarantine during COVID-19 |  |  | < 0.001** |  |  | < 0.001** |  |  | < 0.001** |
| Centralized | 803 (21.2) | 2,985 (78.8) |  | 1,632 (23.4) | 5,357 (76.6) |  | 1,805 (17.8) | 8,320 (82.2) |  |
| At home | 834 (13.4) | 5,385 (86.6) |  | 1,694 (16.5) | 8,600 (83.5) |  | 2,308 (13.6) | 14,689 (86.4) |  |
| None | 3,331 (12.7) | 22,880 (87.3) |  | 2,932 (15.6) | 15,882 (84.4) |  | 1,153 (12.6) | 8,031 (87.4) |  |
| Families/friends hospitalization related to COVID-19 |  |  | 0.36 |  |  | 0.16 |  |  | 0.21 |
| Yes | 774 (14.1) | 4,713 (85.9) |  | 1,623 (17.8) | 7,488 (82.2) |  | 2,426 (14.8) | 14,009 (85.2) |  |
| No | 4,194 (13.6) | 26,537 (86.4) |  | 4,635 (17.2) | 22,351 (82.8) |  | 2,840 (14.3) | 17,031 (85.7) |  |
| Families/friends death related to COVID-19 |  |  | 0.35 |  |  | 0.04* |  |  | < 0.001** |
| Yes | 124 (14.8) | 713 (85.2) |  | 691 (18.5) | 3,040 (81.5) |  | 873 (16.3) | 4,477 (83.7) |  |
| No | 4,844 (13.7) | 30,537 (86.3) |  | 5,567 (17.2) | 26,799 (82.8) |  | 4,393 (14.2) | 26,563 (85.8) |  |
| Psychological intervention during COVID-19 |  |  |  |  |  |  |  |  |  |
| Psychological intervention during COVID-19 |  |  | 0.33 |  |  | 0.12 |  |  | 0.39 |
| Yes | 1,299 (14.0) | 7,967 (86.0) |  | 1,903 (17.8) | 8,775 (82.2) |  | 1,854 (14.7) | 10,740 (85.3) |  |
| No | 3,669 (13.6) | 23,283 (86.4) |  | 4,355 (17.1) | 21,064 (82.9) |  | 3,412 (14.4) | 20,300 (85.6) |  |

The factors with significance in the univariable analyses were then entered into the multivariable logistic regression (refer to **Figure 3** for final factors included in the multivariable model). COVID-19, coronavirus disease 2019; PHQ-9, Patient Health Questionnaire-9; NA, not applicable. **P* < 0.05 (Univariable logistic regression); ***P* < 0.01 (Univariable logistic regression).
