# Supplementary material for: Mental health symptoms and associated factors for general population at the stable, recurrence, and end-of-emergency stages of the COVID-19 pandemic: a repeated national cross-sectional study
Source: Epidemiol Psychiatr Sci. 2025 Oct 14;34:e50. doi: 10.1017/S2045796025100243 (PMC12555081; doi:10.1017/S2045796025100243)
Supplement: Wang et al. supplementary material 4 — Wang et al. supplementary material [file S2045796025100243sup004.docx]

Supplementary Table 4. Univariable logistic regression in exploring potential factors associated with PTSD symptom (measured by IES-R) of all included participants at different pandemic stages (*n_Stable_* = 36,218, *n_Recurrence_* = 36,097, and *n_End-of-emergency_* = 36,306).

|  | Stable stage  (Sampled 2021) | | | Recurrence stage  (Sampled 2022) | | | End-of-emergency stage  (Sampled 2023) | | |
| --- | --- | --- | --- | --- | --- | --- | --- | --- | --- |
| Factor | Scores ≥ 33 | Scores < 33 | *P* value | Scores ≥ 33 | Scores < 33 | *P* value | Scores ≥ 33 | Scores < 33 | *P* value |
| Region division |  |  |  |  |  |  |  |  |  |
| Socio-geographic region (NEPD, normal period) |  |  | 0.26 |  |  | 0.34 |  |  | 0.39 |
| Eastern region | 753 (5.2) | 13,801 (94.8) |  | 1,112 (7.6) | 13,471 (92.4) |  | 1,360 (9.3) | 13,251 (90.7) |  |
| Middle region | 487 (5.4) | 8,489 (94.6) |  | 715 (8.0) | 8,178 (92.0) |  | 853 (9.5) | 8,117 (90.5) |  |
| Western region | 469 (4.8) | 9,290 (95.2) |  | 714 (7.3) | 9,010 (92.7) |  | 856 (8.8) | 8,860 (91.2) |  |
| Northeast region | 144 (4.9) | 2,785 (95.1) |  | 216 (7.5) | 2,681 (92.5) |  | 275 (9.1) | 2,734 (90.9) |  |
| COVID-19 pandemic area I (initial wave, 2020) |  |  | < 0.001** |  |  | 0.004** |  |  | 0.02* |
| Widely infected area (≥ 10,000 confirmed cases) | 116 (7.9) | 1,347 (92.1) |  | 142 (9.7) | 1,323 (90.3) |  | 162 (11.0) | 1,309 (89.0) |  |
| Moderate infected area (≥ 500 confirmed cases) | 1,044 (5.2) | 19,054 (94.8) |  | 1,546 (7.7) | 18,459 (92.3) |  | 1,880 (9.3) | 18,250 (90.7) |  |
| Less infected area (< 500 confirmed cases) | 693 (4.7) | 13,964 (95.3) |  | 1,069 (7.3) | 13,558 (92.7) |  | 1,302 (8.9) | 13,403 (91.1) |  |
| COVID-19 pandemic area II (recurrence, 2022) |  |  | NA |  |  | 0.75 |  |  | 0.71 |
| High risk area (≥ 10,000 confirmed cases) | NA | NA |  | 113 (8.1) | 1,282 (91.9) |  | 137 (9.7) | 1,282 (90.3) |  |
| Moderate risk area (≥ 500 confirmed cases) | NA | NA |  | 1,472 (7.6) | 17,962 (92.4) |  | 1,814 (9.3) | 17,753 (90.7) |  |
| Low risk area (< 500 confirmed cases) | NA | NA |  | 1,172 (7.7) | 14,096 (92.3) |  | 1,393 (9.1) | 13,927 (90.9) |  |
| COVID-19 pandemic area III (end-of-emergency, 2023) |  |  | NA |  |  | NA |  |  | 0.15 |
| Severe affected area (≥ 10,000 confirmed cases) | NA | NA |  | NA | NA |  | 897 (9.7) | 8,376 (90.3) |  |
| Moderate affected area (≥ 5,000 confirmed cases) | NA | NA |  | NA | NA |  | 998 (9.2) | 9,816 (90.8) |  |
| Mild affected area (< 5,000 confirmed cases) | NA | NA |  | NA | NA |  | 1,449 (8.9) | 14,770 (91.1) |  |
| Characteristic |  |  |  |  |  |  |  |  |  |
| Gender |  |  | 0.21 |  |  | 0.06 |  |  | 0.38 |
| Male | 923 (5.0) | 17,633 (95.0) |  | 1,357 (7.4) | 17,035 (92.6) |  | 1,690 (9.1) | 16,921 (90.9) |  |
| Female | 930 (5.3) | 16,732 (94.7) |  | 1,400 (7.9) | 16,305 (92.1) |  | 1,654 (9.3) | 16,041 (90.7) |  |
| Age, years |  |  | 0.36 |  |  | 0.56 |  |  | 0.49 |
| 18-34 | 479 (4.8) | 9,461 (95.2) |  | 727 (7.4) | 9,154 (92.6) |  | 891 (8.9) | 9,136 (91.1) |  |
| 35-49 | 532 (5.1) | 9,892 (94.9) |  | 815 (7.9) | 9,512 (92.1) |  | 982 (9.5) | 9,356 (90.5) |  |
| 50-64 | 519 (5.4) | 9,149 (94.6) |  | 733 (7.7) | 8,816 (92.3) |  | 894 (9.2) | 8,857 (90.8) |  |
| ≥65 | 323 (5.2) | 5,863 (94.8) |  | 482 (7.6) | 5,858 (92.4) |  | 577 (9.3) | 5,613 (90.7) |  |
| Place of residence |  |  | 0.40 |  |  | 0.24 |  |  | 0.13 |
| Urban | 988 (5.0) | 18,668 (95.0) |  | 1,474 (7.5) | 18,208 (92.5) |  | 1,773 (9.0) | 17,934 (91.0) |  |
| Rural | 865 (5.2) | 15,697 (94.8) |  | 1,283 (7.8) | 15,132 (92.2) |  | 1,571 (9.5) | 15,028 (90.5) |  |
| Education level |  |  | 0.51 |  |  | 0.30 |  |  | 0.17 |
| Less than college | 1,438 (5.1) | 26,894 (94.9) |  | 2,138 (7.6) | 26,136 (92.4) |  | 2,605 (9.1) | 26,017 (90.9) |  |
| College degree or higher | 415 (5.3) | 7,471 (94.7) |  | 619 (7.9) | 7,204 (92.1) |  | 739 (9.6) | 6,945 (90.4) |  |
| Marriage status |  |  | 0.77 |  |  | 0.35 |  |  | 0.72 |
| Unmarried | 357 (5.1) | 6,603 (94.9) |  | 521 (7.5) | 6,408 (92.5) |  | 640 (9.1) | 6,383 (90.9) |  |
| Married | 1,342 (5.1) | 25,061 (94.9) |  | 2,005 (7.6) | 24,392 (92.4) |  | 2,426 (9.2) | 23,967 (90.8) |  |
| Divorced/Widowed | 154 (5.4) | 2,701 (94.6) |  | 231 (8.3) | 2,540 (91.7) |  | 278 (9.6) | 2,612 (90.4) |  |
| History of chronic diseases |  |  | 0.72 |  |  | 0.70 |  |  | 0.83 |
| Yes | 176 (5.4) | 3,079 (94.6) |  | 261 (8.0) | 2,997 (92.0) |  | 312 (9.5) | 2,987 (90.5) |  |
| No | 1,618 (5.1) | 30,217 (94.9) |  | 2,416 (7.6) | 29,361 (92.4) |  | 2,935 (9.2) | 28,981 (90.8) |  |
| Unknown | 59 (5.2) | 1,069 (94.8) |  | 80 (7.5) | 982 (92.5) |  | 97 (8.9) | 994 (91.1) |  |
| History of psychiatric disorders |  |  | 0.55 |  |  | 0.49 |  |  | 0.65 |
| Yes | 26 (6.3) | 387 (93.7) |  | 39 (9.1) | 389 (90.9) |  | 45 (10.5) | 384 (89.5) |  |
| No | 1,771 (5.1) | 32,937 (94.9) |  | 2,640 (7.6) | 31,970 (92.4) |  | 3,192 (9.2) | 31,512 (90.8) |  |
| Unknown | 56 (5.1) | 1,041 (94.9) |  | 78 (7.4) | 981 (92.6) |  | 107 (9.1) | 1,066 (90.9) |  |
| Occupation |  |  | 0.67 |  |  | 0.61 |  |  | 0.57 |
| Students, full-time | 74 (4.3) | 1,649 (95.7) |  | 109 (6.5) | 1,572 (93.5) |  | 138 (8.2) | 1,553 (91.8) |  |
| Technicians and associate professionals | 182 (5.1) | 3,418 (94.9) |  | 271 (7.4) | 3,408 (92.6) |  | 312 (8.6) | 3,301 (91.4) |  |
| Government and clerical support workers | 160 (5.0) | 3,069 (95.0) |  | 244 (7.6) | 2,982 (92.4) |  | 302 (9.3) | 2,947 (90.7) |  |
| Social and life service workers | 517 (5.4) | 8,999 (94.6) |  | 761 (8.0) | 8,773 (92.0) |  | 929 (9.6) | 8,755 (90.4) |  |
| Agricultural, forestry and fishery workers | 362 (5.2) | 6,644 (94.8) |  | 532 (7.5) | 6,539 (92.5) |  | 649 (9.1) | 6,448 (90.9) |  |
| Production and manufacture workers | 449 (5.0) | 8,540 (95.0) |  | 685 (7.7) | 8,199 (92.3) |  | 807 (9.2) | 7,986 (90.8) |  |
| Other unclassified occupations | 5 (4.8) | 100 (95.2) |  | 7 (7.2) | 90 (92.8) |  | 9 (8.7) | 94 (91.3) |  |
| Freelance or inoccupation | 104 (5.1) | 1,946 (94.9) |  | 148 (7.7) | 1,777 (92.3) |  | 198 (9.5) | 1,878 (90.5) |  |
| Yearly family income, CNY |  |  | 0.35 |  |  | 0.25 |  |  | 0.11 |
| <40,000 | 398 (5.3) | 7,099 (94.7) |  | 594 (7.9) | 6,943 (92.1) |  | 716 (9.5) | 6,841 (90.5) |  |
| 40,000-99,999 | 1,169 (5.1) | 21,556 (94.9) |  | 1,744 (7.7) | 20,946 (92.3) |  | 2,122 (9.3) | 20,681 (90.7) |  |
| ≥100,000 | 286 (4.8) | 5,710 (95.2) |  | 419 (7.1) | 5,451 (92.9) |  | 506 (8.5) | 5,440 (91.5) |  |
| Activity and work/study status |  |  |  |  |  |  |  |  |  |
| Outside activity/Once |  |  | 0.68 |  |  | 0.39 |  |  | 0.70 |
| 1-7 days | 1,006 (5.2) | 18,224 (94.8) |  | 632 (8.1) | 7,205 (91.9) |  | 2,145 (9.3) | 20,802 (90.7) |  |
| 8-14 days | 556 (5.1) | 10,426 (94.9) |  | 898 (7.5) | 11,006 (92.5) |  | 905 (9.0) | 9,156 (91.0) |  |
| 15-29 days | 189 (4.8) | 3,719 (95.2) |  | 658 (7.4) | 8,262 (92.6) |  | 225 (8.9) | 2,307 (91.1) |  |
| ≥30 days | 102 (4.9) | 1,996 (95.1) |  | 569 (7.7) | 6,867 (92.3) |  | 69 (9.0) | 697 (91.0) |  |
| Work/Study status |  |  | 0.64 |  |  | 0.53 |  |  | 0.74 |
| On-site work/study | 1,155 (5.2) | 21,236 (94.8) |  | 789 (7.9) | 9,212 (92.1) |  | 2,662 (9.2) | 26,185 |  |
| Off-site work/study | 358 (4.9) | 6,943 (95.1) |  | 1,248 (7.5) | 15,333 (92.5) |  | 431 (9.0) | 4,379 |  |
| Not back to work/study | 340 (5.2) | 6,186 (94.8) |  | 720 (7.6) | 8,795 (92.4) |  | 251 (9.5) | 2,398 |  |
| Experience related to COVID-19 |  |  |  |  |  |  |  |  |  |
| Current COVID-19 identity |  |  | 0.08 |  |  | 0.22 |  |  | 0.59 |
| Current infected | 25 (6.2) | 377 (93.8) |  | 339 (7.8) | 3,983 (92.2) |  | 211 (9.3) | 2,046 (90.7) |  |
| Previous infected | 191 (5.7) | 3,158 (94.3) |  | 473 (7.5) | 5,818 (92.5) |  | 2,288 (9.2) | 22,533 (90.8) |  |
| Suspect infected | 37 (6.6) | 520 (93.4) |  | 439 (8.3) | 4,860 (91.7) |  | 272 (9.8) | 2,511 (90.2) |  |
| Not infected | 1,600 (5.0) | 30,310 (95.0) |  | 1,506 (7.5) | 18,679 (92.5) |  | 573 (8.9) | 5,872 (91.1) |  |
| Frontline workers during COVID-19 |  |  | 0.37 |  |  | 0.18 |  |  | 0.06 |
| Yes | 323 (5.3) | 5,715 (94.7) |  | 538 (8.0) | 6,160 (92.0) |  | 778 (9.8) | 7,198 (90.2) |  |
| No | 1,530 (5.1) | 28,650 (94.9) |  | 2,219 (7.5) | 27,180 (92.5) |  | 2,566 (9.1) | 25,764 (90.9) |  |
| Experience of hospitalization for COVID-19 |  |  | 0.45 |  |  | 0.50 |  |  | 0.34 |
| Yes | 147 (5.4) | 2,563 (94.6) |  | 356 (7.9) | 4,159 (92.1) |  | 775 (9.5) | 7,401 (90.5) |  |
| No | 1,706 (5.1) | 31,802 (94.9) |  | 2,401 (7.6) | 29,181 (92.4) |  | 2,569 (9.1) | 25,561 (90.9) |  |
| Experience of quarantine during COVID-19 |  |  | < 0.001** |  |  | < 0.001** |  |  | < 0.001** |
| Centralized | 245 (6.5) | 3,543 (93.5) |  | 739 (10.6) | 6,250 (89.4) |  | 1,137 (11.2) | 8,988 (88.8) |  |
| At home | 323 (5.2) | 5,896 (94.8) |  | 772 (7.5) | 9,522 (92.5) |  | 1,450 (8.5) | 15,547 (91.5) |  |
| None | 1,285 (4.9) | 24,926 (95.1) |  | 1,246 (6.6) | 17,568 (93.4) |  | 757 (8.2) | 8,427 (91.8) |  |
| Families/friends hospitalization related to COVID-19 |  |  | 0.49 |  |  | 0.22 |  |  | 0.09 |
| Yes | 291 (5.3) | 5,196 (94.7) |  | 723 (7.9) | 8,388 (92.1) |  | 1,560 (9.5) | 14,875 (90.5) |  |
| No | 1,562 (5.1) | 29,169 (94.9) |  | 2,034 (7.5) | 24,952 (92.5) |  | 1,784 (9.0) | 18,087 (91.0) |  |
| Families/friends death related to COVID-19 |  |  | 0.08 |  |  | < 0.001** |  |  | < 0.001** |
| Yes | 54 (6.5) | 783 (93.5) |  | 355 (9.5) | 3,376 (90.5) |  | 650 (12.1) | 4,700 (87.9) |  |
| No | 1,799 (5.1) | 33,582 (94.9) |  | 2,402 (7.4) | 29,964 (92.6) |  | 2,694 (8.7) | 28,262 (91.3) |  |
| Psychological intervention during COVID-19 |  |  |  |  |  |  |  |  |  |
| Psychological intervention during COVID-19 |  |  | 0.42 |  |  | 0.65 |  |  | 0.10 |
| Yes | 489 (5.3) | 8,777 (94.7) |  | 805 (7.5) | 9,873 (92.5) |  | 1,117 (8.9) | 11,477 (91.1) |  |
| No | 1,364 (5.1) | 25,588 (94.9) |  | 1,952 (7.7) | 23,467 (92.3) |  | 2,227 (9.4) | 21,485 (90.6) |  |

The factors with significance in the univariable analyses were then entered into the multivariable logistic regression (refer to **Figure 3** for final factors included in the multivariable model). COVID-19, coronavirus disease 2019; PTSD, post-traumatic stress disorder; IES-R, Impact of Events Scale-Revised; NA, not applicable. **P* < 0.05 (Univariable logistic regression); ***P* < 0.01 (Univariable logistic regression).
