# Supplementary material for: Mental health symptoms and associated factors for general population at the stable, recurrence, and end-of-emergency stages of the COVID-19 pandemic: a repeated national cross-sectional study
Source: Epidemiol Psychiatr Sci. 2025 Oct 14;34:e50. doi: 10.1017/S2045796025100243 (PMC12555081; doi:10.1017/S2045796025100243)
Supplement: Wang et al. supplementary material 5 — Wang et al. supplementary material [file S2045796025100243sup005.docx]

Supplementary Table 5. Univariable logistic regression in exploring potential independent factors associated with insomnia symptom (measured by ISI) of all included participants at different pandemic stages (*n_Stable_* = 36,218, *n_Recurrence_* = 36,097, and *n_End-of-emergency_* = 36,306).

|  | Stable stage  (Sampled 2021) | | | Recurrence stage  (Sampled 2022) | | | End-of-emergency stage  (Sampled 2023) | | |
| --- | --- | --- | --- | --- | --- | --- | --- | --- | --- |
| Factor | Scores ≥ 15 | Scores < 15 | *P* value | Scores ≥ 15 | Scores < 15 | *P* value | Scores ≥ 15 | Scores < 15 | *P* value |
| Region division |  |  |  |  |  |  |  |  |  |
| Socio-geographic region (NEPD, normal period) |  |  | 0.20 |  |  | 0.13 |  |  | 0.17 |
| Eastern region | 2,393 (16.4) | 12,161 (83.6) |  | 3,298 (22.6) | 11,285 (77.4) |  | 2,789 (19.1) | 11,822 (80.9) |  |
| Middle region | 1,519 (16.9) | 7,457 (83.1) |  | 1,996 (22.4) | 6,897 (77.6) |  | 1,677 (18.7) | 7,293 (81.3) |  |
| Western region | 1,541 (15.8) | 8,218 (84.2) |  | 2,081 (21.4) | 7,643 (78.6) |  | 1,744 (17.9) | 7,972 (82.1) |  |
| Northeast region | 472 (16.1) | 2,457 (83.9) |  | 632 (21.8) | 2,265 (78.2) |  | 558 (18.5) | 2,451 (81.5) |  |
| COVID-19 pandemic area I (initial wave, 2020) |  |  | 0.37 |  |  | 0.49 |  |  | 0.25 |
| Widely infected area (≥ 10,000 confirmed cases) | 255 (17.4) | 1,208 (82.6) |  | 328 (22.4) | 1,137 (77.6) |  | 274 (18.6) | 1,197 (81.4) |  |
| Moderate infected area (≥ 500 confirmed cases) | 3,308 (16.5) | 16,790 (83.4) |  | 4,391 (21.9) | 15,614 (78.1) |  | 3,693 (18.3) | 16,437 (81.7) |  |
| Less infected area (< 500 confirmed cases) | 2,362 (16.1) | 12,295 (83.9) |  | 3,288 (22.5) | 11,339 (77.5) |  | 2,801 (19.0) | 11,904 (81.0) |  |
| COVID-19 pandemic area II (recurrence, 2022) |  |  | NA |  |  | 0.01* |  |  | 0.18 |
| High risk area (≥ 10,000 confirmed cases) | NA | NA |  | 346 (24.8) | 1,049 (75.2) |  | 281 (19.8) | 1,138 (80.2) |  |
| Moderate risk area (≥ 500 confirmed cases) | NA | NA |  | 4,354 (22.4) | 15,080 (77.6) |  | 3,691 (18.9) | 15,876 (81.1) |  |
| Low risk area (< 500 confirmed cases) | NA | NA |  | 3,307 (21.7) | 11,961 (78.3) |  | 2,796 (18.3) | 12,524 (81.7) |  |
| COVID-19 pandemic area III (end-of-emergency, 2023) |  |  | NA |  |  | NA |  |  | 0.08 |
| Severe affected area (≥ 10,000 confirmed cases) | NA | NA |  | NA | NA |  | 1,789 (19.3) | 7,484 (80.7) |  |
| Moderate affected area (≥ 5,000 confirmed cases) | NA | NA |  | NA | NA |  | 2,032 (18.8) | 8,782 (81.2) |  |
| Mild affected area (< 5,000 confirmed cases) | NA | NA |  | NA | NA |  | 2,947 (18.2) | 13,272 (81.8) |  |
| Characteristic |  |  |  |  |  |  |  |  |  |
| Gender |  |  | 0.47 |  |  | 0.23 |  |  | 0.07 |
| Male | 3,010 (16.2) | 15,546 (83.8) |  | 4,032 (21.9) | 14,360 (78.1) |  | 3,403 (18.3) | 15,208 (81.7) |  |
| Female | 2,915 (16.5) | 14,747 (83.5) |  | 3,975 (22.5) | 13,730 (77.5) |  | 3,365 (19.0) | 14,330 (81.0) |  |
| Age, years |  |  | 0.56 |  |  | 0.23 |  |  | 0.40 |
| 18-34 | 1,583 (15.9) | 8,357 (84.1) |  | 2,123 (21.5) | 7,758 (78.5) |  | 1,827 (18.2) | 8,200 (81.8) |  |
| 35-49 | 1,716 (16.5) | 8,708 (83.5) |  | 2,325 (22.5) | 8,002 (77.5) |  | 1,951 (18.9) | 8,387 (81.1) |  |
| 50-64 | 1,610 (16.7) | 8,058 (83.3) |  | 2,157 (22.6) | 7,392 (77.4) |  | 1,856 (19.0) | 7,895 (81.0) |  |
| ≥65 | 1,016 (16.4) | 5,170 (83.6) |  | 1,402 (22.1) | 4,938 (77.9) |  | 1,134 (18.3) | 5,056 (81.7) |  |
| Place of residence |  |  | 0.72 |  |  | 0.20 |  |  | 0.70 |
| Urban | 3,203 (16.3) | 16,453 (83.7) |  | 4,315 (21.9) | 15,367 (78.1) |  | 3,688 (18.7) | 16,019 (81.3) |  |
| Rural | 2,722 (16.4) | 13,840 (83.6) |  | 3,692 (22.5) | 12,723 (77.5) |  | 3,080 (18.6) | 13,519 (81.4) |  |
| Education level |  |  | 0.56 |  |  | 0.35 |  |  | 0.65 |
| Less than college | 4,618 (16.3) | 23,714 (83.7) |  | 6,241 (22.1) | 22,033 (77.9) |  | 5,322 (18.6) | 23,300 (81.4) |  |
| College degree or higher | 1,307 (16.6) | 6,579 (83.4) |  | 1,766 (22.6) | 6,057 (77.4) |  | 1,446 (18.8) | 6,238 (81.2) |  |
| Marriage status |  |  | 0.66 |  |  | 0.39 |  |  | 0.11 |
| Unmarried | 1,139 (16.4) | 5,821 (83.6) |  | 1,544 (22.3) | 5,385 (77.7) |  | 1,323 (18.8) | 5,700 (81.2) |  |
| Married | 4,302 (16.3) | 22,101 (83.7) |  | 5,821 (22.1) | 20,576 (77.9) |  | 4,867 (18.4) | 21,526 (81.6) |  |
| Divorced/Widowed | 484 (17.0) | 2,371 (83.0) |  | 642 (23.2) | 2,129 (76.8) |  | 578 (20.0) | 2,312 (80.0) |  |
| History of chronic diseases |  |  | 0.34 |  |  | 0.29 |  |  | 0.39 |
| Yes | 562 (17.3) | 2,693 (82.7) |  | 758 (23.3) | 2,500 (76.7) |  | 644 (19.5) | 2,655 (80.5) |  |
| No | 5,177 (16.3) | 26,658 (83.7) |  | 7,013 (22.1) | 24,764 (77.9) |  | 5,919 (18.5) | 25,997 (81.5) |  |
| Unknown | 186 (16.5) | 942 (83.5) |  | 236 (22.2) | 826 (77.8) |  | 205 (18.8) | 886 (81.2) |  |
| History of psychiatric disorders |  |  | 0.53 |  |  | 0.37 |  |  | 0.08 |
| Yes | 76 (18.4) | 337 (81.6) |  | 107 (25.0) | 321 (75.0) |  | 98 (22.8) | 331 (77.2) |  |
| No | 5,671 (16.3) | 29,037 (83.7) |  | 7,666 (22.1) | 26,944 (77.9) |  | 6,450 (18.6) | 28,254 (81.4) |  |
| Unknown | 178 (16.2) | 919 (83.8) |  | 234 (22.1) | 825 (77.9) |  | 220 (18.8) | 953 (81.2) |  |
| Occupation |  |  | 0.58 |  |  | 0.72 |  |  | 0.90 |
| Students, full-time | 268 (15.6) | 1,455 (84.4) |  | 351 (20.9) | 1,330 (79.1) |  | 302 (17.9) | 1,389 (82.1) |  |
| Technicians and associate professionals | 568 (15.8) | 3,032 (84.2) |  | 798 (21.7) | 2,881 (78.3) |  | 663 (18.4) | 2,950 (81.6) |  |
| Government and clerical support workers | 528 (16.4) | 2,701 (83.6) |  | 738 (22.9) | 2,488 (77.1) |  | 607 (18.7) | 2,642 (81.3) |  |
| Social and life service workers | 1,621 (17.0) | 7,895 (83.0) |  | 2,146 (22.5) | 7,388 (77.5) |  | 1,853 (19.1) | 7,831 (80.9) |  |
| Agricultural, forestry and fishery workers | 1,126 (16.1) | 5,880 (83.9) |  | 1,551 (21.9) | 5,520 (78.1) |  | 1,307 (18.4) | 5,790 (81.6) |  |
| Production and manufacture workers | 1,466 (16.3) | 7,523 (83.7) |  | 1,969 (22.2) | 6,915 (77.8) |  | 1,628 (18.5) | 7,165 (81.5) |  |
| Other unclassified occupations | 15 (14.3) | 90 (85.7) |  | 19 (19.6) | 78 (80.4) |  | 18 (17.5) | 85 (82.5) |  |
| Freelance or inoccupation | 333 (16.2) | 1,717 (83.8) |  | 435 (22.6) | 1,490 (77.4) |  | 390 (18.8) | 1,686 (81.2) |  |
| Yearly family income, CNY |  |  | 0.80 |  |  | 0.46 |  |  | 0.24 |
| <40,000 | 1,240 (16.5) | 6,257 |  | 1,704 (22.6) | 5,833 (77.4) |  | 1,445 (19.1) | 6,112 (80.9) |  |
| 40,000-99,999 | 3,719 (16.4) | 19,006 |  | 5,029 (22.2) | 17,661 (77.8) |  | 4,254 (18.7) | 18,549 (81.3) |  |
| ≥100,000 | 966 (16.1) | 5,030 |  | 1,274 (21.7) | 4,596 (78.3) |  | 1,069 (18.0) | 4,877 (82.0) |  |
| Activity and work/study status |  |  |  |  |  |  |  |  |  |
| Outside activity/Once |  |  | < 0.001** |  |  | 0.004** |  |  | 0.47 |
| 1-7 days | 3,055 (15.9) | 16,175 (84.1) |  | 1,739 (22.2) | 6,098 (77.8) |  | 4,279 (18.6) | 18,668 (81.4) |  |
| 8-14 days | 1,765 (16.1) | 9,217 (83.9) |  | 2,550 (21.4) | 9,354 (78.6) |  | 1,851 (18.4) | 8,210 (81.6) |  |
| 15-29 days | 667 (17.1) | 3,241 (82.9) |  | 1,959 (22.0) | 6,961 (78.0) |  | 480 (19.0) | 2,052 (81.0) |  |
| ≥30 days | 438 (20.9) | 1,660 (79.1) |  | 1,759 (23.7) | 5,677 (76.3) |  | 158 (20.6) | 608 (79.4) |  |
| Work/Study status |  |  | 0.47 |  |  | 0.41 |  |  | 0.66 |
| On-site work/study | 3,651 (16.3) | 18,740 (83.7) |  | 2,263 (22.6) | 7,738 (77.4) |  | 5,367 (18.6) | 23,480 (81.4) |  |
| Off-site work/study | 1,175 (16.1) | 6,126 (83.9) |  | 3,636 (21.9) | 12,945 (78.1) |  | 890 (18.5) | 3,920 (81.5) |  |
| Not back to work/study | 1,099 (16.8) | 5,427 (83.2) |  | 2,108 (22.2) | 7,407 (77.8) |  | 511 (19.3) | 2,138 (80.7) |  |
| Experience related to COVID-19 |  |  |  |  |  |  |  |  |  |
| Current COVID-19 identity |  |  | 0.46 |  |  | < 0.001** |  |  | 0.047* |
| Current infected | 69 (17.2) | 333 (82.8) |  | 943 (21.8) | 3,379 (78.2) |  | 430 (19.1) | 1,827 (80.9) |  |
| Previous infected | 568 (17.0) | 2,781 (83.0) |  | 1,320 (21.0) | 4,971 (79.0) |  | 4,631 (18.7) | 20,190 (81.3) |  |
| Suspect infected | 101 (18.1) | 456 (81.9) |  | 1,636 (30.9) | 3,663 (69.1) |  | 562 (20.2) | 2,221 (79.8) |  |
| Not infected | 5,187 (16.3) | 26,723 (83.7) |  | 4,108 (20.4) | 16,077 (79.6) |  | 1,145 (17.8) | 5,300 (82.2) |  |
| Frontline workers during COVID-19 |  |  | < 0.001** |  |  | < 0.001** |  |  | < 0.001** |
| Yes | 1,093 (18.1) | 4,945 (81.9) |  | 1,766 (26.4) | 4,932 (73.6) |  | 1,693 (21.2) | 6,283 (78.8) |  |
| No | 4,832 (16.0) | 25,348 (84.0) |  | 6,241 (21.2) | 23,158 (78.8) |  | 5,075 (17.9) | 23,255 (82.1) |  |
| Experience of hospitalization for COVID-19 |  |  | 0.40 |  |  | 0.17 |  |  | 0.39 |
| Yes | 459 (16.9) | 2,251 (83.1) |  | 1,037 (23.0) | 3,478 (77.0) |  | 1,551 (19.0) | 6,625 (81.0) |  |
| No | 5,466 (16.3) | 28,042 (83.7) |  | 6,970 (22.1) | 24,612 (77.9) |  | 5,217 (18.5) | 22,913 (81.5) |  |
| Experience of quarantine during COVID-19 |  |  | 0.02* |  |  | < 0.001** |  |  | < 0.001** |
| Centralized | 677 (17.9) | 3,111 (82.1) |  | 1,859 (26.6) | 5,130 (73.4) |  | 2,047 (20.2) | 8,078 (79.8) |  |
| At home | 1,026 (16.5) | 5,193 (83.5) |  | 2,214 (21.5) | 8,080 (78.5) |  | 3,091 (18.2) | 13,906 (81.8) |  |
| None | 4,222 (16.1) | 21,989 (83.9) |  | 3,934 (20.9) | 14,880 (79.1) |  | 1,630 (17.7) | 7,554 (82.3) |  |
| Families/friends hospitalization related to COVID-19 |  |  | 0.80 |  |  | 0.12 |  |  | 0.49 |
| Yes | 904 (16.5) | 4,583 (83.5) |  | 2,075 (22.8) | 7,036 (77.2) |  | 3,089 (18.8) | 13,346 (81.2) |  |
| No | 5,021 (16.3) | 25,710 (83.7) |  | 5,932 (22.0) | 21,054 (78.0) |  | 3,679 (18.5) | 16,192 (81.5) |  |
| Families/friends death related to COVID-19 |  |  | 0.25 |  |  | 0.02* |  |  | < 0.001** |
| Yes | 149 (17.8) | 688 (82.2) |  | 885 (23.7) | 2,846 (76.3) |  | 1,203 (22.5) | 4,147 (77.5) |  |
| No | 5,776 (16.3) | 29,605 (83.7) |  | 7,122 (22.0) | 25,244 (78.0) |  | 5,565 (18.0) | 25,391 (82.0) |  |
| Psychological intervention during COVID-19 |  |  |  |  |  |  |  |  |  |
| Psychological intervention during COVID-19 |  |  | 0.49 |  |  | 0.30 |  |  | 0.65 |
| Yes | 1,537 (16.6) | 7,729 (83.4) |  | 2,406 (22.5) | 8,272 (77.5) |  | 2,364 (18.8) | 10,230 (81.2) |  |
| No | 4,388 (16.3) | 22,564 (83.7) |  | 5,601 (22.0) | 19,818 (78.0) |  | 4,404 (18.6) | 19,308 (81.4) |  |

The factors with significance in the univariable analyses were then entered into the multivariable logistic regression (refer to **Figure 3** for final factors included in the multivariable model). COVID-19, coronavirus disease 2019; ISI, Insomnia Severity Index; NA, not applicable. **P* < 0.05 (Univariable logistic regression); ***P* < 0.01 (Univariable logistic regression).
